# Splenic differentiation and emergence of CCR5^+^CXCL9^+^CXCL10^+^ monocyte-derived dendritic cells in the brain during cerebral malaria

**DOI:** 10.1038/ncomms13277

**Published:** 2016-11-03

**Authors:** Isabella C. Hirako, Marco A. Ataide, Lucas Faustino, Patricia A. Assis, Elizabeth W. Sorensen, Hisashi Ueta, Natalia M. Araújo, Gustavo B. Menezes, Andrew D. Luster, Ricardo T. Gazzinelli

**Affiliations:** 1 Centro de Pesquisas René Rachou, Fundação Oswaldo Cruz, Av. Augusto de Lima 1715, Barro Preto, Belo Horizonte MG 30190-002, Brazil; 2 Division of Rheumatology, Allergy and Immunology, Massachusetts General Hospital, Harvard Medical School, 149 Thirteenth Street, Charlestown, Massachusetts 02129, USA; 3 Departamento de Bioquímica e Imunologia and Centro de Biologia Gastrointestinal, Departamento de Morfologia, Universidade Federal of Minas Gerais, Av. Antonio Carlos 6627, Belo Horizonte MG 31270-901, Brazil; 4 Division of Infectious Diseases and Immunology, University of Massachusetts Medical School, 364 Plantation Street, Worcester, Massachusetts 01655, USA

## Abstract

Dendritic cells have an important role in immune surveillance. After being exposed to microbial components, they migrate to secondary lymphoid organs and activate T lymphocytes. Here we show that during mouse malaria, splenic inflammatory monocytes differentiate into monocyte-derived dendritic cells (MO-DCs), which are CD11b^+^F4/80^+^CD11c^+^MHCII^high^DC-SIGN^high^Ly6c^+^ and express high levels of CCR5, CXCL9 and CXCL10 (CCR5^+^CXCL9/10^+^ MO-DCs). We propose that malaria-induced splenic MO-DCs take a reverse migratory route. After differentiation in the spleen, CCR5^+^CXCL9/10^+^ MO-DCs traffic to the brain in a CCR2-independent, CCR5-dependent manner, where they amplify the influx of CD8^+^ T lymphocytes, leading to a lethal neuropathological syndrome.

## Abstract (web summary)

Cerebral malaria is an often fatal complication of *Plasmodium* infection involving accumulation of inflammatory leukocytes in the central nervous system. Here the authors map the development and trafficking of CCR5^+^ monocyte-derived dendritic cells from the spleen to the brains of *Plasmodium berghei* ANKA infected mice.

Monocytes, macrophages and dendritic cells (DCs) are heterogeneous cell populations that have critical roles in tissue repair, sensing the presence of invasive microorganisms and initiating protective immune responses. These cell subsets have overlapping functions. DCs are more specialized in antigen presentation and shaping T-cell-mediated immunity, whereas macrophages primarily act as a source of proinflammatory cytokines and phagocytic cells that effectively destroy pathogens. Monocytes are less specialized cells that contribute to the overall production of inflammatory cytokines, anti-microbial effector functions and are the main progenitors for DCs and macrophages^1^,^2^,^3^.

DCs, monocytes and macrophages are thought to have an important role in host resistance to both mouse and human malaria^4^,^5^. During malaria, DCs are activated through Toll-like receptors (TLRs), primarily TLR9 (refs 6, 7, 8, 9), and serve as an important source of interleukin (IL)-12. IL-12 activates natural killer cells to produce interferon-γ (IFNγ) and promotes differentiation of T-helper type 1 (Th1) lymphocytes that orchestrate acquired protective immunity against *Plasmodium* infection^10^,^11^,^12^,^13^,^14^,^15^,^16^. Importantly, uptake of infected erythrocytes seems to inhibit maturation and function of human DCs^17^, and a low number of circulating DCs is associated with impairment of antigen-specific T-cell responses in symptomatic patients infected with either *Plasmodium falciparum* or *Plasmodium vivax*, suggesting their role in resistance to human disease^18^. In addition, inflammatory monocytes in mouse (CCR2^+^Ly6c^+^ and F4/80^+^) and humans (CD14^+^CD16^high^), as well as in DCs, are important sources of inflammatory cytokines and reactive nitrogen and oxygen intermediates that effectively destroy *Plasmodium* parasites within phagocytosed infected red blood cells (iRBCs)^11^,^19^,^20^,^21^.

DCs also contribute to the pathogenesis of mouse malaria. Blockade of T cell and DC interaction prevents a deleterious response that is associated with a wasting syndrome and hypothermia in *Plasmodium chabaudi-*infected mice^22^. DCs are also required for the development of experimental cerebral malaria (ECM)^23^. In addition, a subset of Ly6c^+^ monocytic cells is critical for recruiting CD8^+^ T cells to the central nervous system (CNS) and development of ECM^24^. Furthermore, a high proportion of inflammatory monocytes and DCs express inflammasomes and on a secondary microbial stimuli release excessive levels of IL-1β, both in mouse and human malaria^25^.

Although most DC progenitors that enter peripheral tissues at homeostasis are not monocytes, studies have demonstrated that in response to infection or inflammation, monocytes can be induced to differentiate and provide important DC functions^26^,^27^,^28^,^29^,^30^,^31^,^32^,^33^,^34^,^35^,^36^. Monocyte-derived DCs (MO-DCs) share many morphological and functional characteristics with conventional DCs (cDCs), including antigen capture and presentation to CD4^+^ T lymphocytes, as well cross-presentation to CD8^+^ T lymphocytes. Monocytes are more abundant than DCs in the blood and bone marrow (BM), and the *in vivo* mobilization of this monocyte reservoir to generate potent antigen-presenting DCs is of central importance during microbial infection^31^,^32^,^33^,^34^,^36^.

Studies have defined markers that enable the distinction of cDCs and inflammatory monocytes from MO-DCs; however, the role of MO-DCs in mouse malaria, as well as in neuroinflammation observed during ECM, has not been explored. Here we report that MO-DCs emerge as a main splenic DC population during early stages of *Plasmodium berghei* ANKA (PbA) infection in mice. These MO-DCs are unique in that they express high levels of the chemokine receptor CCR5, as well as the IFN-inducible CXCR3 chemokine ligands CXCL9 (MIG) and CXCL10 (IP10) (CCR5^+^CXCL9/10^+^ MO-DCs). CCR5^+^CXCL9/10^+^ MO-DCs are the main DC subset in the CNS of mice with cerebral malaria. Importantly, emergence of MO-DCs in the CNS and development of ECM is dependent on MO-DC CCR5 expression and independent of CCR2 expression. Our results reveal a previously unappreciated role of MO-DCs in PbA-induced neuroinflammation and the mechanism by which CCR5 mediates the development of ECM.

## Results

### Malaria infection induces MO-DCs

**Figure 1:**
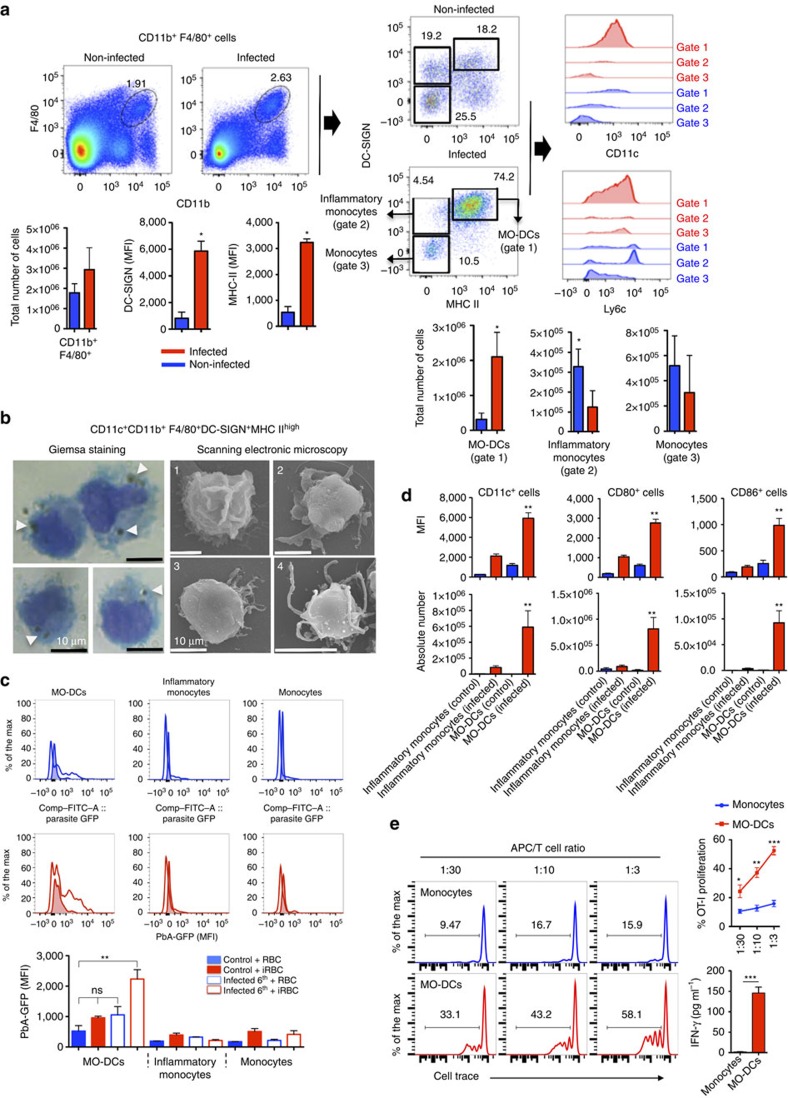
Differentiation of splenic MO-DCs in PbA-infected mice. Spleens were harvested 5 days after PbA infection. (**a**) Splenocytes were first gated for F4/80^+^CD11b^+^ cells and then for DC-SIGN^+^MHC II^high^ cells. F4/80^+^CD11b^+^DC-SIGN^high^MHC II^high^ cells were checked for CD11c^+^ and Ly6c^+^ expression. Bar graphs correspond to total number of cells (four mice per group). The data shown are representative of eight independent experiments. Results are expressed as median±s.d. An asterisk indicates that difference is statistically significant when comparing infected and non-infected mice (*P*<0.01) after Mann–Whitney *U*-test. (**b**) Splenocytes were labelled with fluorescent antibodies and CD11c^+^CD11b^+^F4/80^+^DC-SIGN^high^MHC II^high^ MO-DCs purified by flow cytometry and analysed by Giemsa staining and optical microscopy, as well as SEM. Panel 1 and panels 2, 3 and 4 of SEM illustrate MO-like and DC-like cells, respectively. White arrows indicate haemozoin crystals inside purified MO-DCs (scale bar, 10 μm). (**c**) Flow cytometry histograms show the MFI results from spleen cells of uninfected (shaded lines) versus infected (open lines) incubated, at 1:1 ratio, with uninfected RBCs (blue) or RBCs infected with GFP-expressing PbA (red). The results indicate the phagocytic activity of MO-DCs, inflammatory monocytes and monocytes, as indicated. Bar graphs correspond to the MFI average of three to four mice per group. The data shown are representative of two independent experiments. (**d**) Expression of co-stimulatory molecules CD80 and CD86 on cell surface of monocytes, inflammatory monocytes and MO-DCs. (**e**) Cell Trace-labelled naive OT-I cells were cultured with monocytes or MO-DCs (>95% purity) from PbA-infected mice at indicated APC/T cell ratio in the presence of OVA protein (40 μg ml^−1^), and 3 days later the percentage of Cell Trace^low^ CD8^+^ T cells was evaluated. Representative histograms on the left and top right graph show average and s.e. of T-cell proliferation. Average and s.e. of IFNγ production in the medium of co-cultures at 1:3 ratio measured by enzyme-linked immunosorbent assay are shown in the right bottom graph. Data are representative of two independent experiments with three mice per group, which yielded similar results. Results expressed as mean±s.d. Differences considered statistically significant comparing infected and non-infected mice after two-way analysis of variance, *P<0.05, **P<0.01.

Recent studies have demonstrated that *in vivo* microbial challenge signal inflammatory monocytes to differentiate into MO-DCs^35^,^36^. Here we evaluated whether MO-DCs emerge during mouse malaria by searching for CD11c^+^MHC II^high^CD11b^+^F4/80^+^DC-SIGN^high^ cells in the spleen, a main site of phagocytic cell interaction with *Plasmodium* iRBCs. For this purpose, we gated double-positive CD11b and F4/80 spleen cells for MHC II^high^DC-SIGN^+^CD11c^+^ (ref. 35). The results presented in Fig. 1a indicate that the frequency of MO-DCs in total CD11b^+^F4/80^+^ splenic cells was increased from 18% in uninfected to 74% in PbA-infected mice. In addition, the level of expression, as indicated by the mean fluorescence intensity (MFI), of DC-SIGN and major histocompatibility complex (MHC) II in MO-DCs from infected mice increased threefold. A fraction of these cells also expressed different levels of Ly6c. In contrast, the frequency of CD11b^+^F4/80^+^DC-SIGN^int^MHC II^−^CD11c^−^Ly6c^high^ cells (inflammatory monocytes) decreased from 19% to 4.4%, suggesting that inflammatory monocytes were converted into MO-DCs. After infection, most monocytes (Gate 3, CD11b^+^F4/80^+^DC-SIGN^−^MHC II^−^CD11c^−^) became Ly6c^high^, but as a whole the difference in number of cells was not statistically significant when comparing uninfected with infected wild-type (WT) mice. We also performed the initial gating on CD11c^+^MHC II^high^ cells and then on the DC-SIGN^+^LY6c^+^ population, and confirmed that over 89% of these cells in PbA-infected mice were CD11b^+^F4/80^+^ (Supplementary Fig. 1A). In addition, our analysis indicated that the frequency of CD11c^+^MHC II^high^CD11b^−^F4/80^−^DC^−^SIGN^−^Ly6c^−^ cells, which correspond to cDCs, decreased from 48% in uninfected control mice to 20% of total CD11c^+^MHC II^high^ in infected mice.

To further characterize the MO-DCs, we sorted MO-DCs (95–99% purity) by flow cytometry (Supplementary Fig. 2) and analysed by Giemsa staining. and optical and scanning electronic microscopy (SEM) (Fig. 1b). The results obtained from SEM show that morphology of MO-DCs is heterogeneous. Although a significant proportion of MO-DCs from PbA-infected mice were typical DCs with a smooth surface and dendrites, other cells displayed characteristics of monocytes or transitional morphological phenotypes. Interestingly, we found in the Giemsa staining that a high frequency of MO-DCs purified from infected mice contained haemozoin, which are haem polymers produced by the parasite. To further evaluate the phagocytic capacity of MO-DCs, we incubated spleen cells from uninfected controls and infected mice with erythrocytes infected with a PbA clone that expresses green fluorescent protein (GFP). By flow cytometry, we demonstrated that MO-DCs were highly efficient phagocytic cells, when compared with inflammatory monocytes and monocytes (Fig. 1c). In addition, we observed that unlike inflammatory monocytes and monocytes, MO-DCs express very high levels of CD80 and CD86 (Fig. 1d and Supplementary Fig. 1B). Furthermore, MO-DCs, but not monocytes, cross-presented soluble antigens (Fig. 1e), suggesting that they play an important role on activating CD8^+^ T lymphocytes either in the spleen or in the CNS.

### Malaria-induced CCR5^+^CXCL9/10^+^ MO-DCs

**Figure 2:**
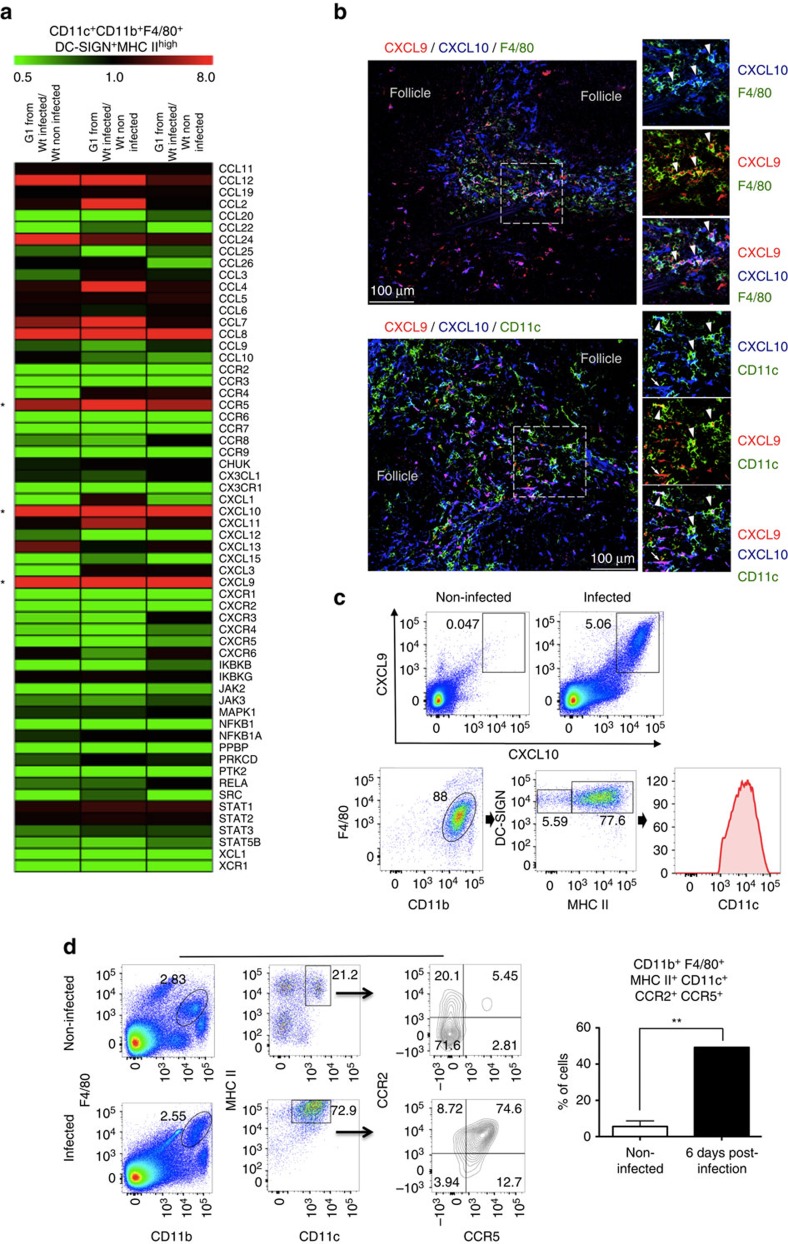
Expression of CXCL9 and CXCL10 by MO-DCs from PbA-infected mice. Splenocytes were harvested at 6 days post PbA infection. CD11c^+^CD11b^+^F4/80^+^DC-SIGN^high^MHC II^high^ MO-DCs were purified by flow cytometry (Supplementary Fig. 2) and analysed for (**a**) RNA expression of immune-related genes using an nCounter Analysis System. The results indicate fold increase of gene expression, when comparing MO-DCs isolated from three infected mice compared with three non-infected mice. (**b**) Confocal microscopy of the spleen from an infected REX3 mouse. Top panels shows F4/80^+^ (APC converted in green), CXCL9-RFP and CXCL10-BFP staining. Bottom panels show CD11c^+^ (APC converted in green), CXCL9-RFP and CXCL10-BFP staining. Arrows and arrowheads show double- and triple-positive cells, respectively (scale bar, 100 μm). (**c**) PbA-infected REX3 mice were killed at day 6 post infection and splenic MO-DC cells analysed for CXCL9 and CXCL10 expression. Spleen cells were first gated on double-positive CXCL9/CXCL10, and then on F4/80^+^CD11b^+^ and DC-SIGN^+^MHC II^+^ cells. All CXCL9^+^CXCL10^+^F4/80^+^CD11b^+^DC-SIGN^+^MHC II^+^ cells were CD11c^+^. (**d**) Spleen cells harvested 6 days post infection were first gated on F4/80^+^CD11b^+^ and then on CD11c^+^MHC II^+^ cells. The majority of MO-DCs from uninfected and PbA-infected mice were CCR2^+^CCR5^−^ and CCR2^+^CCR5^+^, respectively. Representative primary flow cytometric dot plots are shown. The data are representative of three experiments with three mice per group that yielded similar results. Results are expressed as median±s.d. Difference considered statistically significant when comparing infected and non-infected mice (**P>0.01) after Mann–Whitney *U*-test.

To further characterize MO-DCs, lysates of highly purified fluorescence-activated cell-sorted MO-DCs (Supplementary Fig. 2) were obtained and gene transcripts analysed for 547 immune-related genes plus 14 housekeeping genes using a commercially available set of nanostring probes. We observed an increase in the expression of ∼18% of the analysed genes in MO-DCs isolated from the spleens of infected mice compared with uninfected mice. Of note, the induction in the expression of *CXCL9* (24-fold increase) and *CXCL10* (19-fold increase) stood out. In addition, the expression of *CCR5* was enhanced sixfold (Fig. 2a). As CXCL9 and CXCL10 have been shown to play an important role on ECM^37^,^38^,^39^, we further investigated the expression of these chemokines in spleens of PbA-infected mice. The results presented in Supplementary Fig. 3A show that expression of *CXCL9* and *CXCL10* messenger RNA achieved the highest level from 5 to 7 days post infection, coinciding with the peak of MO-DCs in the spleen. We also used the reporting expression of CXCR3 ligands (REX3) transgenic mice that express the red fluorescent protein (RFP) and blue fluorescent protein (BFP) as reporter genes under the control of the *CXCL9* and *CXCL10* promoters, respectively^40^. We found that a large proportion of the double-positive RFP^+^BFP^+^ cells from infected mice were MO-DCs (Fig. 2b,c). Likewise, over 80% of these cells were CCR2^+^CCR5^+^, whereas the inflammatory monocytes and MO-DCs from uninfected mice were CCR2^+^, but CCR5^−^ (Fig. 2d).

**Figure 3:**
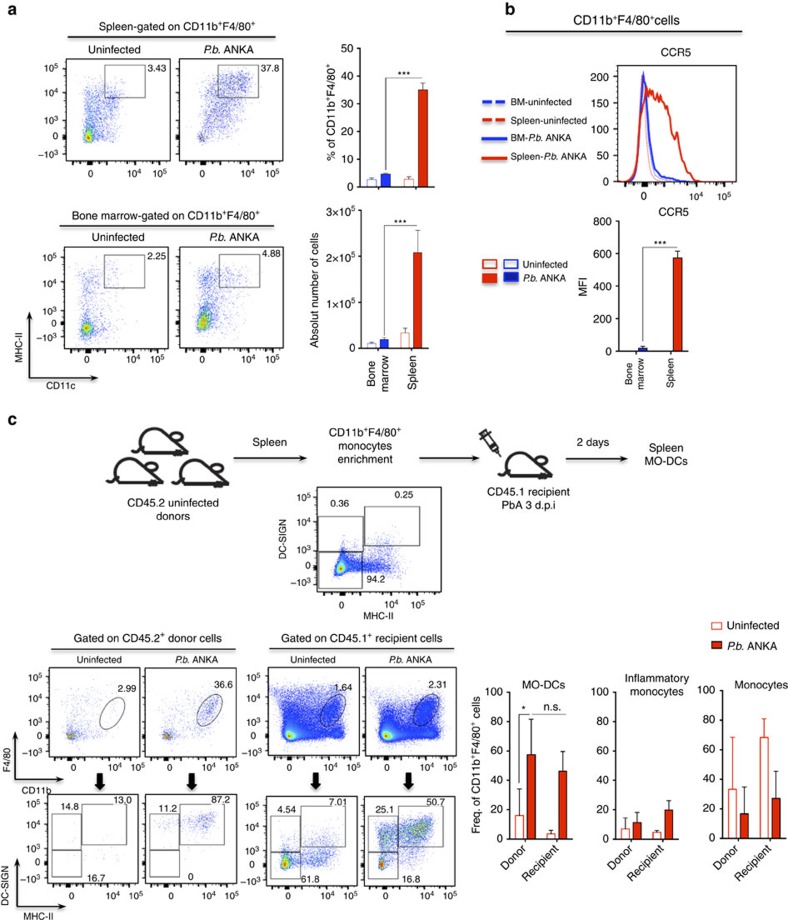
Differentiation of splenic MO-DCs in mice infected with PbA. Spleens and BM were harvested from mice, either uninfected or 5 days post PbA infection. Splenocytes and BM cells were then labelled and analysed by fluorescence-activated cell sorting. We first gated F4/80^+^CD11b^+^ cells and then CD11c^+^MHC II^high^ cells, which are the MO-DCs. (**a**) Representative primary flow cytometric dot plots are shown. Upper bar graph on the right corresponds to the frequency of CD11c^+^MHC II^high^ cells and botton bar graph to the total number of CD11c^+^MHC II^high^ cells within the total F4/80^+^CD11b^+^ population. (**b**) Flow cytometry histograms show the MFI results of CCR5 expression in total F4/80^+^CD11b^+^ cells of uninfected (shaded lines) versus infected (open lines). Bar graph corresponds to MFI average of CCR5 expression. The data shown in **a** and **b** are representative of two independent experiments (three mice per group). (**c**) Enriched splenic F4/80^+^CD11b^+^DC-SIGN^−^MHC-II^−^ cells from uninfected CD45.2^+^ donor mice were adoptively transferred i.v. into CD45.1^+^ uninfected or PbA-infected mice. Dot plot of enriched monocytes show that <0.5% of the cells were CD11c^+^ MHC II^high^ (MO-DCs). After 48 h, spleens of recipient mice were obtained and frequency of CD45.2^+^ donor-derived MO-DC, inflammatory monocytes and monocytes were compared with corresponding populations of CD45.1^+^ recipient mice. Bar graphs show frequency of MO-DC, inflammatory monocytes and monocytes in F4/80^+^CD11b^+^ populations from CD45.2^+^ or CD45.1^+^ cells of two pooled experiments that yielded similar results. Results are expressed as mean±s.d. Differences were considered statistically significant when *P<0.05, as indicated by two-way analysis of variance, ***P<0.001.

We also asked whether MO-DCs might be directly recruited from the BM. WT mice were infected with PbA and emergence of inflammatory monocytes or MO-DCs in the BM and spleens evaluated side-by-side. Our results presented in Fig. 3a,b indicate that there is an increase of F4/80^+^, CD11b^+^ and MHCII^+^ in the BM. However, in contrast to spleen MO-DCs, these cells were both CD11C^−^ and CCR5^−^. Hence, our results suggest that inflammatory monocytes acquire the MO-DC phenotype elsewhere and cannot be recruited directly from the BM by CCR5 ligands. Importantly, we performed the adoptive cell transfer experiments. Monocytes (F4/80^+^CD11b^+^DC^−^SIGN^−^MHCII^−^CD11c^−^) were enriched from spleens of uninfected CD45.2 C57BL/6 mice and two million cells transferred to either uninfected or PbA-infected CD45.1 congenic mice. Two days later, we gated F4/80^+^CD11b^+^ cells and looked for distribution of DC-SIGN^−^MHCII^−^, DC-SIGN^+^MHCII^−^ and DC-SIGN^+^MHCII^+^, which were either CD45.2^+^ or CD45.1^+^. We found that the frequencies of splenic CD45.2^+^ MO, inflammatory monocytes and MO-DCS, as well as splenic CD45.1^+^ MO, inflammatory monocytes and MO-DCs, were similar, respectively (Fig. 3c). In uninfected mice, we found that migration of F4/80^+^CD11b^+^ CD45.2^+^ MO to the spleen was highly reduced and we detected very low frequency of either CD45.1^+^ MO-DCs or CD45.2^+^ MO-DCs. These data are consistent with the hypothesis that splenic monocytes are differentiating into MO-DCs.

### CCR5^+^CXCL9/10^+^ MO-DCs in the CNS of PbA-infected mice

**Figure 4:**
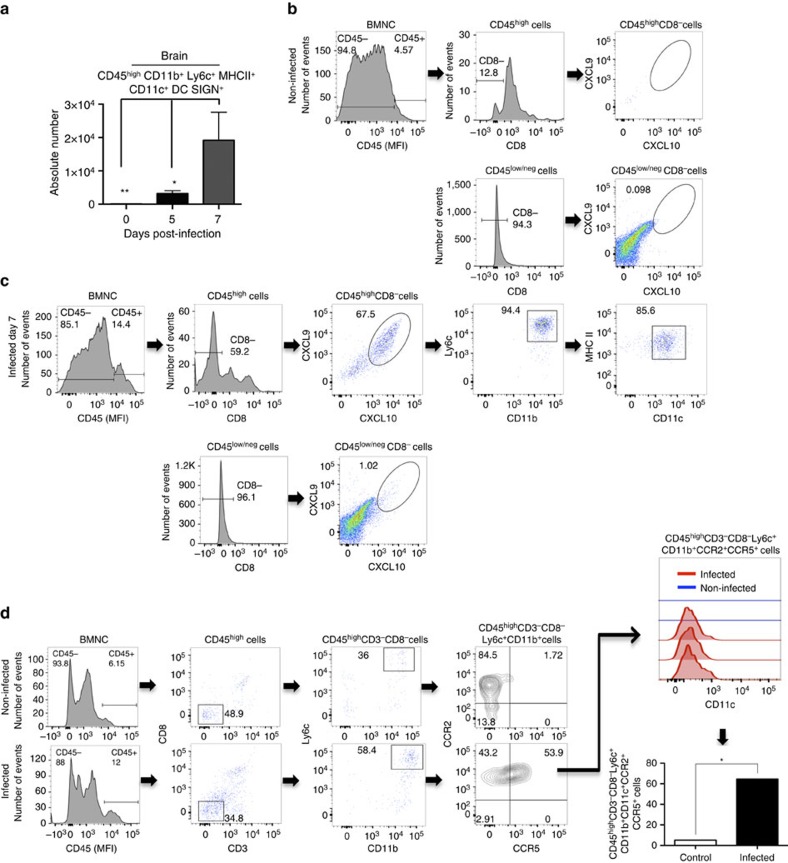
High levels of MO-DCs in the brain from PbA-infected mice. (**a**) MO-DCs (CD45^high^CD8^−^CD3^−^CD11b^+^Ly6c^+^MHC II^+^DC-SIGN^high^CD11c^+^) were quantified by flow cytometric analysis within brain mononuclear cells (BMNCs) isolated at 0, 5 and 7 days post infection with PbA. (**b**) BMNCs from uninfected REX3 mice were analysed by flow cytometric analysis. Cells that were first selected on CD45^high^ and then CD8^−^ cells were negative for CXCL9 (RFP) and CXCL10 (RFP). (**c**) Cells first selected on CD45^high^ and then CD8^−^ from BMNCs from PbA-infected REX3 mice were ∼70% double positive for the CXCL9 and CXCL10 reporters. Eighty percent of CD45^hi^CD8^−^CXCL9^+^CXCL10^+^ cells were CD11b^+^Ly6c^+^CD11c^+^MHC II^+^. CD45^low/negative^CD8^−^ BMNCs were all negative for CXCL9-RFP and CXCL10-BFP. Representative primary flow cytometric dot plots are shown after CD45^high^ gating for leukocytes. (**d**) BMNCs were harvested from WT mice at 0 and 7 days post infection and expression of CCR2 and CCR5 analysed in MO-DCs, first selected on CD45^high^, gated on CD8^−^CD3^−^ and then on Ly6c^+^CD11b^+^. The histogram in the far right represents the MFI of CD11c expression on MO-DCs from uninfected and PbA-infected mice. Data represent the average of three or four mice per group and results are representative of three independent experiments. Differences were considered statistically significant when *P<0.05 or **P<0.01 as indicated by two-way analysis of variance (values are means±s.d.). (**a**) or Mann–Whitney *U*-test (values are medians±s.d.) (**d**).

We next evaluated the presence of MO-DCs in the CNS of mice undergoing ECM. To analyse the presence of MO-DCs in the CNS of PbA-infected mice, we first gated the CD45^high^ brain mononuclear cells to discriminate haematopoietic cells from microglia (CD45^interm^). As the cellular infiltrate in ECM is highly enriched for CD8^+^ T cells, we then gated on CD45^high^CD8^−^ cells. MO-DCs emerge at day 5 and peaked at day 7 post infection with PbA, which coincides with the expression of CXCL9 and CXCL10, and other inflammatory mediators in the CNS (Fig. 4a and Supplementary Figs 3B and 4A). Importantly, most of CD45^+^CD11c^+^MHC II^high^ cells in the CNS were also CD11b^+^F4/80^+^, which is consistent with the MO-DC phenotype. We further analysed brain mononuclear cells from PbA-infected mice for CXCL9, CXCL10 and CCR5 expression. We found no cells expressing CXCL9 and CXCL10 in uninfected REX3 mice (Fig. 4b). In infected REX3 mice, BFP^+^RFP^+^ cells were CD45^high^. CD45^high^CD8^+^ cells were BFP^−^RFP^−^, whereas ∼60% of CD45^high^CD8^−^ cells were BFP^+^RFP^+^ (Fig. 4c). Furthermore, 80% of CD45^high^CD8^−^BFP^+^RFP^+^ cells were CD11b^+^Ly6c^+^CD11c^+^ MHC II^high^, consistent with the hypothesis that MO-DCs are also a main haematopoietic source of CXCL9 and CXCL10 in the CNS of infected mice (Fig. 4c). CD45^interm^ and CD45^−^ cells, most probably microglia and neuron or glial cells, respectively, were both BFP and RFP negative. Finally, we evaluated the expression of CCR2 and CCR5 in MO-DCs derived from CNS of infected mice. The results shown in Fig. 4d demonstrate that ∼60% of brain MO-DCs from infected, but not from control mice, were double positive for CCR2 and CCR5.

### IFNγ-induced expression of CXCL9 and CXCL10 by MO-DCs

**Figure 5:**
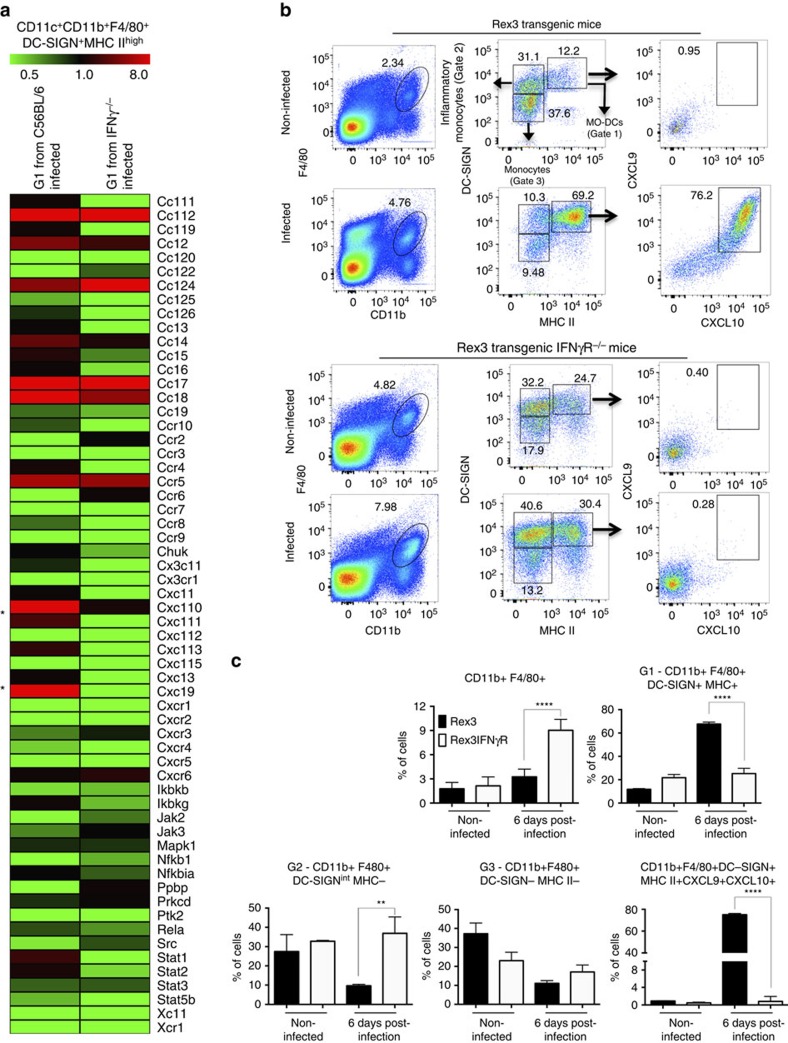
IFNγ-dependent expression of CXCL9 and CXCL10 by MO-DCs from PbA-infected mice. C57BL/6, IFNγ^−/−^, REX3 or REX3/IFNγR^−/−^ mice were infected with PbA and their spleens harvested at 6 days later. (**a**) Gene expression was analysed in total RNA extracted from highly purified (over 98% purity) MO-DCs from infected C57BL/6 and IFNγ^−/−^ mice by nCounter Analysis System. Data represent the average of gene expression of three mice per group. (**b**) Representative primary flow cytometric dot plots are shown for splenic MO-DCs from REX3 and REX3/IFNγR^−/−^ mice gated on F4/80^+^CD11b^+^ (left panels), then on DC-SIGN^high^MHC II^high^ cells (middle panels), and CXCL9 and CXCL10 double-positive cells (right panels), respectively. The percentage of MO-DC (Gate 1), inflammatory monocytes (Gate 2) and monocytes (Gate 3), and CXCL9^+^CXCL10^+^MO-DCs are indicated. (**c**) Frequency of splenic CD11b^+^F4/80^+^ cell subsets in infected REX3 and REX3/IFNγR^−/−^ mice are shown. The average and s.d. are representative of two independent experiments with three or four mice per group. Differences were considered statistically significant when **P<0.01 and ****P<0.0001 as indicated by two-way analysis of variance analysis.

A recent study has demonstrated that IFNγ is an important mediator of MO-DC differentiation^36^. In addition, IFNγ has been shown to induce the expression of CXCL9 and CXCL10 in macrophages. Hence, we used IFNγ^−/−^ and REX3IFNγR^−/−^ mice infected with PbA. We first evaluated the profile of gene expression in highly purified splenic MO-DCs (Supplementary Fig. 2) isolated from infected C57BL/6 and *IFNγ*^−/−^ mice. From 98 PbA-induced genes, 58 (60%) were dependent on endogenous IFNγ. Expression of *CXCL9* and *CXCL10* mRNA induced by PbA infection was totally absent in MO-DCs from IFNγ^−/−^ mice, whereas enhanced expression of other genes, such as *CCR5*, *CCL7* and *CCL8*, was independent of endogenous IFNγ (Fig. 5a). Consistently, MO-DCs from REX3, but not from REX3IFNγR^−/−^ mice, expressed high levels of RFP and BFP (Fig. 5b,c).

We also tested whether IFNγ is necessary for differentiation of MO-DCs. We noticed an accumulation of inflammatory monocytes and decreased frequency of MO-DCs in REX3/IFNγR^−/−^ and *IFNγ*^−/−^ relative to REX3 and C57BL/6 mice infected with PbA (Fig. 5b,c and Supplementary Fig. 5). Together, these results indicate that differentiation of precursor inflammatory monocytes into MO-DCs is impaired in mice lacking either functional *IFNγ* or *IFNγR* genes.

### TLR-induced expression of CXCL9 and CXCL10 by MO-DCs

Cheong *et al*.^35^ demonstrated that lipopolysaccharide induces differentiation of inflammatory monocytes into MO-DCs. It is also well established that *Plasmodium* infection activates the innate immune system primarily by activation of TLR9 (refs 6, 7, 8, 9, 41) and other nucleic acid sensors^42^,^43^. The activation of TLR9 during PbA infection induces the production of IL-12 by DCs, culminating in the production of IFNγ, primarily T lymphocytes^6^,^22^,^41^. Hence, we decided to evaluate whether PbA infection was inducing differentiation of MO-DCs via TLR activation. With this in mind, we used E6446, an antagonist that prevents activation of TLR7 and TLR9 by single-stranded RNA and DNA^41^,^44^. The results presented in Supplementary Fig. 6 show that therapy with E6446 prevented lethality when given in the very early days of infection (from day −1 to 3 post infection).

**Figure 6:**
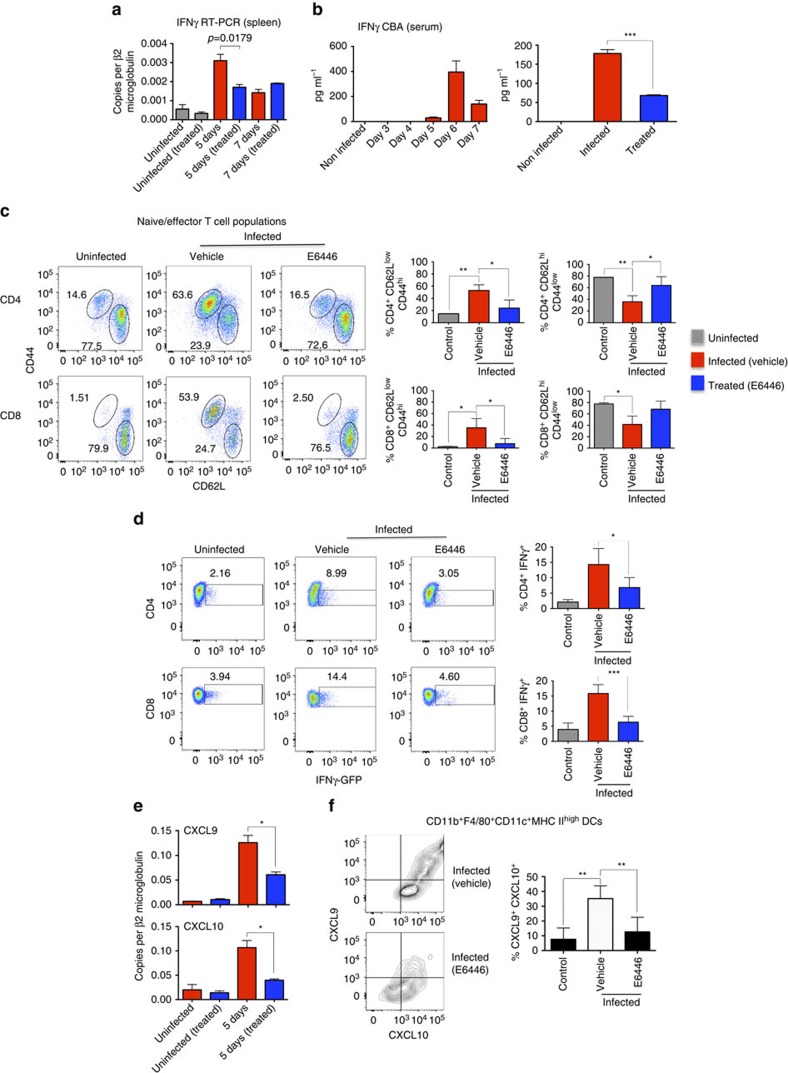
E6446 inhibits expression of IFNγ by T cells and CXCL9 and CXCL10 by MO-DCs. C57BL/6, GREAT and REX3 mice were infected with PbA and treated with either E6446 (120 mg kg^−1^ per day) or vehicle from −1 to 3 days post infection. (**a**) Spleens were harvested from WT mice at 0, 5 and 7 post infection, total RNA extracted and expression levels of IFNγ mRNA analysed by quantitative PCR. The results were normalized to *β2-MICROBULIN*. (**b**) Sera from infected C57BL/6 mice were collected at different times post infection and used to measure the levels of IFNγ by Cytometric Bead Analysis. The inhibitory effect of E6446 on the levels of circulating IFNγ was evaluated at 6 days post infection (right panel). (**c**) Spleens from untreated and E6446-treated C57BL6 mice were harvested 5 days post infection. Cells were gated on CD44 and CD62L from CD4^+^ or CD8^+^. Bar graphs correspond to the percentage of naive (CD62L^high^CD44^low^) versus activated effector (CD62L^low^CD44 ^high^) CD4^+^ T (top panels) and CD8^+^ T (bottom panels) cells. (**d**) Splenocytes from uninfected and infected GREAT mice treated with E6446 or left untreated were stimulated with PMA (50 ng ml^−1^) and ionomycin (500 ng ml^−1^) for 4 h in culture containing brefeldin A and analysed by flow cytometry gated on CD4^+^ or CD8^+^ cells. YFP-positive cells were considered as IFNγ producers CD4^+^ T (top panels) and CD8^+^ T (bottom panels) cells. (**e**) Quantitative reverse transcriptase–PCR (left panels) and (**f**) flow cytometry analysis revealed that treatment with E6446 resulted in inhibition of CXCL9 and CXCL10 mRNA expression in the spleen and RFP and BFP proteins by splenic MO-DCs from PbA-infected C57BL/6 and REX3 mice, respectively. Representative primary flow cytometry dot plots are shown in **c**–**f**. Results are representative of two to three independent experiments with three to five mice per group. Differences considered statistically significant when *P<0.05, **P<0.01, ***P<0.001 or ****P<0.0001, as indicated by two-way analysis of variance analysis (values are means±s.d.).

The data presented in Fig. 6a,b show that treatment with E6446 inhibits the expression of *IFNγ* mRNA by spleen cells and results in decreased circulating levels of IFNγ in sera of mice at 6 days post infection with PbA. In addition, at 5 days post infection, the proportion of activated (CD44^high^/CD62L^low^) CD4^+^ T and CD8^+^ T lymphocytes was augmented to 55.5% and 39.9%, when compared with 14.7% and 12.2% in uninfected controls or 27.8% and 10.2% in infected mice treated with E6446, respectively (Fig. 6c). The percentage of resting T cells (CD44^low^/CD62L^high^) was inversely proportional to activated cells and decreased to 32.9% of CD4^+^ T and 37.3% of CD8^+^ T lymphocytes in infected mice, as compared with 77.7% and 78.1% in uninfected controls and 59.6% and 64% in infected mice treated with E6446, respectively. Likewise, E6446 inhibited the expression of IFNγ by both CD4^+^ T and CD8^+^ T cells from infected mice. The results presented in Fig. 6d were obtained by using GREAT mice that express a *YFP* gene under the IFNγ promoter. Expression of yellow fluorescent protein (YFP) by CD4^+^ T and CD8^+^ T cells isolated from controls and infected mice increased from 2.16 to 8.99% and from 3.94% to 14.40%, respectively. In GREAT mice infected with PbA and treated with E6446, only 3.05% and 4.60% of CD4^+^ T and CD8^+^ T cells expressed YFP, respectively.

As a consequence of inhibition of IFNγ production by T lymphocytes, treatment with E6446 also inhibited the expression of *CXCL9* and *CXCL10* mRNA by spleen cells (Fig. 6e), as well as RFP (CXCL9) and BFP (CXCL10) by MO-DCs from infected WT and REX3 mice, respectively (Fig. 6f). Immunocytochemistry analysis revealed the inhibitory effect of E6446 treatment on CXCL9 and CXCL10 expression by spleen cells from *PbA*-infected REX3 mice (Supplementary Fig. 6B).

### IFNγ-dependent recruitment of MO-DCs to the CNS

**Figure 7:**
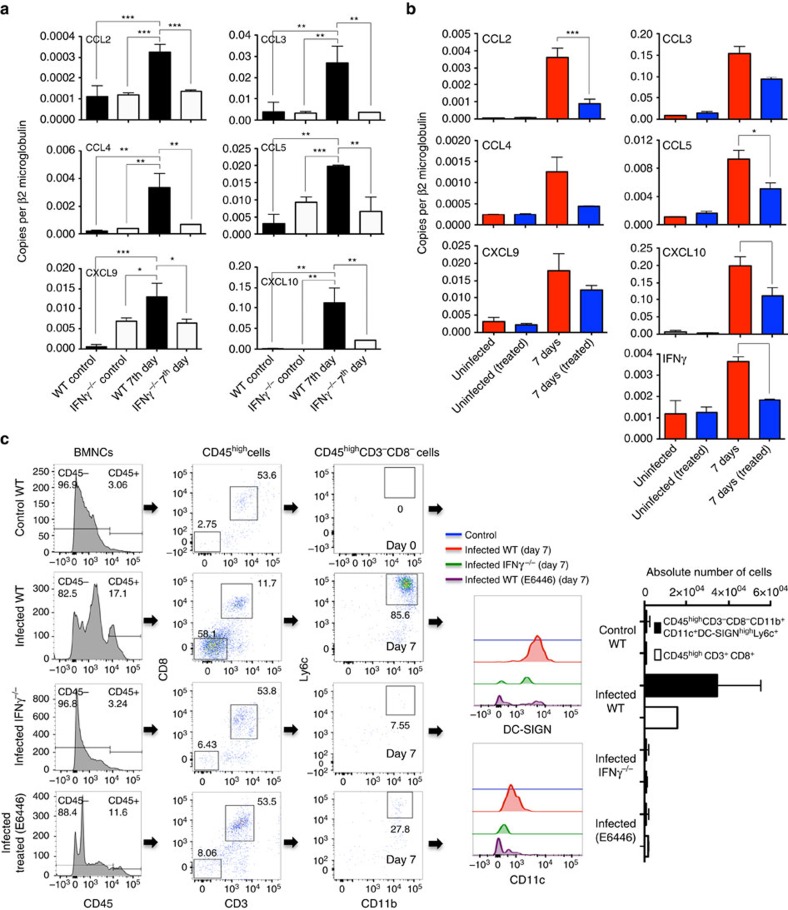
IFNγ-dependent chemokine expression and emergence of CD45^high^ haematopoietic cells. *CCL2*, *CCL3*, *CCL4*, *CCL5*, *CXCL9*, *CXCL10* and *IFNγ* mRNA expression was analysed in brain tissue of uninfected or PbA-infected (**a**) IFNγ^−/−^ and C57BL/6 mice, as well as (**b**) C57BL/6 mice treated from day −1 to 3 post infection with vehicle or E6446 (120 mg kg^−1^ per day), as indicated. The mRNA levels were analysed by quantitative PCR and results presented after normalization with β2-microglobulin mRNA. (**c**) Flow cytometric analysis of brain MO-DCs first selected on CD45high, gated on CD3^−^CD8^−^ and then CD11b^+^Ly6c^+^ (CD11c^+^DC-SIGN^+^) from uninfected and PBA-infected WT, IFNγ^−/−^ and WT mice treated with E6446 7 days post infection. The numbers of both CD45^high^CD8^+^CD3^+^ and CD45^high^CD8^−^CD3^−^CD11c^+^CD11b^+^DC-SIGN^high^Ly6c^+^ cells are significantly decreased in the brain of infected IFNγ^−/−^ and E6446-treated mice. Differences considered statistically significant when *P<0.05, **P<0.01, ***P<0.001 and ****P<0.0001 as indicated by two-way analysis of variance analysis (values are means±s.d.).

The results presented in Supplementary Fig. 3B show that expression of *IFNγ* and chemokine genes (that is, *CCL2*, *CCL3*, *CCL4*, *CCL5*, *CCL8*, *CXCL9* and *CXCL10*) is induced and peaks in the brain at 6–7 days postinfection with PbA. Our experiments also demonstrated that expression of these genes is not seen or largely decreased in the brains of PbA-infected IFNγ^−/−^ mice or PbA-infected C57BL/6 mice treated with E6446 (Fig. 7a,b). As MO-DCs express both CCR2 (receptor for CCL2–MCP1) and CCR5 (receptor for CCL3–MIP1α, CCL4–MIP1β and CCL5–RANTES), we evaluated the emergence of MO-DCs in the brain of PbA-infected *IFNγ*^−/−^ mice and infected C57BL/6 mice treated with E6446. We observed that migration of MO-DCs into the brain of infected IFNγ^−/−^ mice or E6446-treated WT mice was also impaired (Fig. 7c). Consistent with the importance of MO-DCs in recruitment of CD8^+^ T cells and development of ECM^37^,^38^,^39^, the total number of CD8^+^ T lymphocytes was reduced, but not absent, in the CNS of either *IFNγ*^−/−^ mice or PbA-infected C57BL/6 mice treated with E6446. Based on these results and those published elsewhere^4^,^45^,^46^,^47^,^48^, we propose that IFNγ is a key cytokine mediating ECM by also promoting the recruitment of MO-DCs into the brain and consequently amplifying recruitment of CD8^+^ T cells to the CNS.

### CCR5-dependent recruitment of MO-DCs to the CNS

**Figure 8:**
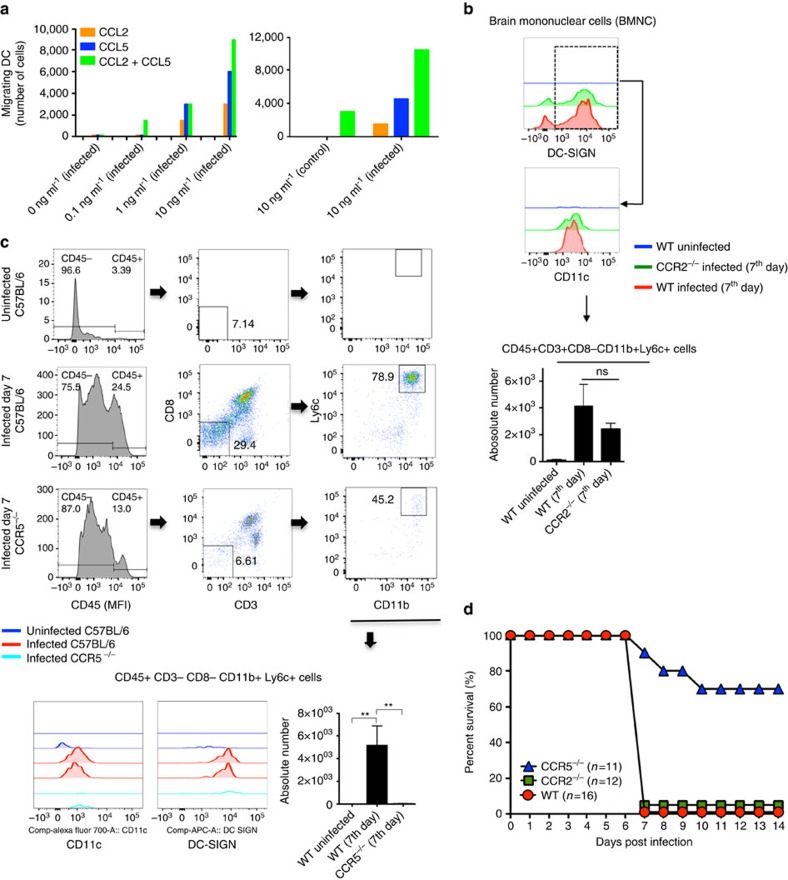
Enhanced resistance of CCR5^−/−^ mice to development of ECM. (**a**) *In vitro* migration of enriched MO-DCs towards different concentrations of CCL2, CCL5 or the combination of CCL2 and CCL5. (**b**) Flow cytometric analysis of MO-DCs in the CNS of WT and CCR2^−/−^ mice infected with PbA. A lower, but not significant difference in the frequency of CD45^high^CD11c^+^CD11b^+^DC-SIGN^high^MHCII^high^Ly6c^+^ is observed in the brain of PbA-infected CCR2^−/−^ mice. (**c**) Representative primary flow cytometric dot plots gated on CD8^−^CD3^−^ and then Ly6c^+^CD11b^+^ cells are shown after gating the CD45^high^ cells. The numbers of CD45^high^CD8^−^CD3^−^CD11c^+^CD11b^+^DC-SIGN^high^Ly6c^+^ are significantly decreased in the brain of PbA-infected CCR5^−/−^ mice. (**d**) Survival curve of PbA-infected C57BL/6, CCR2^−/−^ and CCR5^−/−^ mice. The results are pool of three independent experiments with three to six mice per group. Differences were considered statistically significant when *P<0.01 as indicated by one-way analysis of variance (values are means±s.d.). The survival curves were analysed by log-rank test and CCR5^−/−^ mice were shown more resistant to PbA infection, when compared with CCR2^−/−^ or WT mice (**P<0.01).

We hypothesized that CCL2, CCL3, CCL4 and CCL5 are largely responsible for recruiting MO-DCs to the CNS of PbA-infected mice. The data presented in Fig. 8a provided evidence that isolated MO-DCs (75–85% purity) migrate towards both CCL2 and CCL5 *in vitro*, although CCL5 appeared to be a more efficacious chemoattractant for MO-DCs. We also noticed an additive effect of the two chemokines. As CCL2 and CCR2 have been shown to be important for recruitment of inflammatory monocytes during infection with *Toxoplasma gondii* and *P. chabaudi*^19^,^49^, we evaluated the role of CCR2 in the development of ECM. As expected, we observed a decreased frequency of MO-DCs in the spleen of both uninfected and PbA-infected CCR2^−/−^ mice (Supplementary Fig. 7A,B). However, the number of MO-DCs in the CNS of PbA-infected CCR2^−/−^ mice was only marginally reduced (Fig. 8c). In contrast, we observed no difference in the frequency or number of splenic MO-DCs in infected CCR5^−/−^ mice (Supplementary Fig. 8A,B). Furthermore, we observed no impairment on activation and IFNγ production by CD4^+^T and CD8^+^T cells in spleens from CCR5^−/−^ mice infected with PbA (Supplementary Fig. 9A,B). Nevertheless, emergence of MO-DCs in the CNS of PbA-infected *CCR5*^−/−^ mice was highly compromised (Fig. 8c). Consistent with numbers of MO-DCs in the CNS, CCR5^−/−^ mice, but not CCR2^−/−^ mice, were more resistant to the development of ECM than PbA-infected WT mice (Fig. 8d).

**Figure 9:**
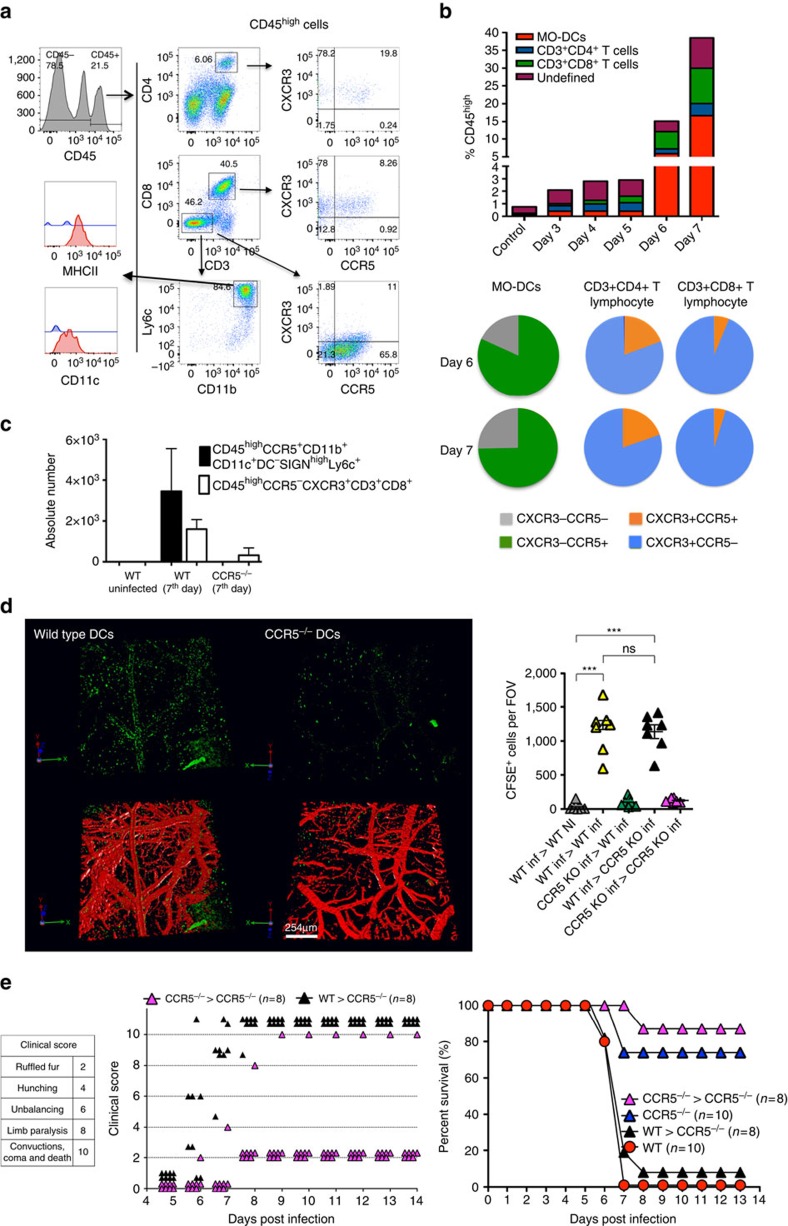
CCR5^+^MO-DCs migrate to the CNS and mediate ECM in PbA-infected mice. (**a**) Brain mononuclear cells (BMNC) were recovered from PbA-infected mice gated on CD45^high^, then on CD3^+^CD8^+^ or CD3^+^CD4^+^ and analysed for CXCR3 and CCR5 expression. Alternatively, CD45^high^ cells were gated on CD3^−^CD8^−^CD11b^+^Ly6c^+^MHC II^+^CD11c^+^ and then analysed for CXCR3 and CCR5 expression. (**b**) Top graph shows the frequency of CD45^high^ cells (that is, MO-DCs, CD4^+^ T and CD8^+^ T cells) in total BMNCs isolated from C57BL/6 mice at different time post infection. Bottom panel shows the frequency of MO-DCs, CD4^+^ T cells and CD8^+^ T cells expressing CXCR3 and CCR5. (**c**) Numbers of both CD45^high^CD8^−^CD3^−^CCR5^+^CD11c^+^CD11b^+^DC-SIGN^high^Ly6c^+^ and CD45^high^CCR5^−^CXCR3^+^CD8^+^CD3^+^ are significantly decreased in the brain of PbA-infected CCR5^−/−^ mice. These results are representative of four experiments (values are means±s.d.). (**d**) MO-DC-enriched populations were obtained from WT and CCR5^−/−^ mice 5 days post infection and labelled with CSFE transferred i.v. (1–2 million cells per mouse) in the orbital vein into PbA-infected WT or CCR5^−/−^ mice. As control, cells were also transferred to uninfected mice. The presence of CSFE labeled cells were detected in the microvasculature and brain parenchyma by confocal microscopy 15–60 min after cell transfer (Supplementary Movies 1–6). Each point represents total number of labelled cells in four field of views per mouse (scale bar, 254 μm). The results are pool of three independent experiments with two or three mice per group. Differences were considered statistically significant when ***P<0.0001, as indicated by one-way analysis of variance analysis. (**e**) Three million splenic MO-DCs derived from either WT or CCR5^−/−^ mice at 5 days post infection were transferred to CCR5^−/−^ mice at four days post infection. Scores of disease severity and survival curves of PbA-infected CCR5^−/−^ mice that received WT MO-DCs or CCR5^−/−^ MO-DCs are shown in left and right panels, respectively. PbA-infected WT or CCR5^−/−^ mice that received no MO-DCs were used as controls. The results are pool of two independent experiments with four mice per group. The survival curves were analysed by log-rank test. PbA-infected CCR5^−/−^ mice that received WT MO-DCs were more susceptible to infection, when compared with PbA-infected CCR5^−/−^ mice that received CCR5^−/−^ MO-DCs or PbA-infected CCR5^−/−^ mice that received no MO-DCs (**P<0.01, ***P<0.001).

Importantly, over 80% of MO-DCs in the CNS of infected C57BL/6 mice expressed CCR5. Less than 10% of CD4^+^ T and CD8^+^ T lymphocytes expressed CCR5, whereas over 99% expressed CXCR3 (Fig. 9a,b). Hence, we hypothesize that CCR5 is a key receptor for MO-DC recruitment to the CNS, whereas CXCR3 and its ligands, CXCL9 and CXCL10, are responsible for attracting T lymphocytes to the CNS. Consistently, there is a decrease in the absolute number of both MO-DCs and CD8^+^ T cells in the CNS of PbA-infected CCR5^−/−^ mice (Fig. 9c). We also performed *in vivo* imaging experiments to evaluate the role of CCR5 in the recruitment of MO-DCs to the CNS. MO-DCs (75–85% purity) from spleens of PbA-infected WT or CCR5^−/−^ mice were transferred into WT or CCR5^−/−^ mice at 5 days post infection. Migration of carboxyfluorescein succinimidyl ester (CSFE)-labelled MO-DCs was evaluated from 15 to 60 min after cell transfer. We found high frequency of WT MO-DCs, primarily in the microvasculature. MO-DCs from CCR5^−/−^ mice were less efficient in migrating to the CNS of infected mice. Furthermore, WT MO-DCs, but not CCR5^−/−^ MO DCs, migrated to the CNS of CCR5^−/−^, indicating that expression of CCR5 is critical for MO-DC migration to the CNS (Fig. 9d and Supplementary Movies 1–6). No migration of MO-DCs to the CNS of uninfected mice was observed. Finally, the results presented in Fig. 9e show that transfer of enriched WT MO-DC population restored ECM in PbA-infected CCR5^−/−^ mice.

## Discussion

**Figure 10:**
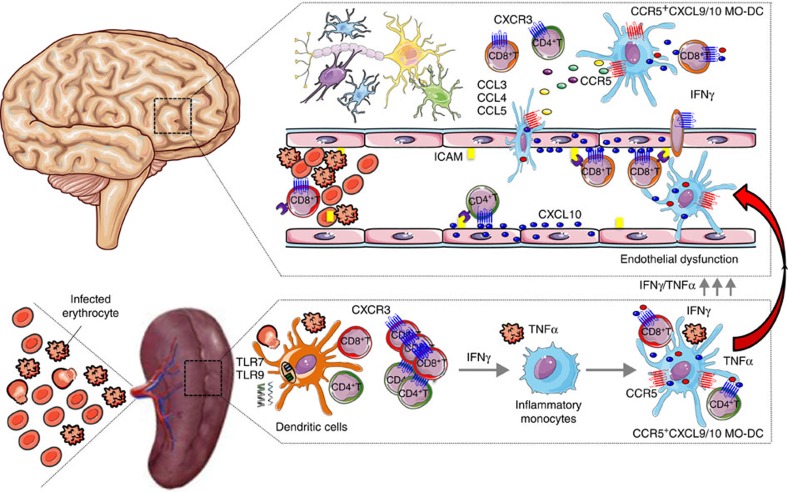
CCR5^+^CXCL9/10^+^ MO-DCs mediate cerebral malaria in PbA-infected mice. Infection with PbA leads to activation of DCs and induces IFNγ-dependent differentiation of inflammatory monocytes into splenic MO-DCs that express high levels of CCR5, CXCL9 and CXCL10 (CCR5^+^CXCL9/10^+^ MO-DCs). We hypothesize that after differentiation, CCR5^+^CXCL9/10^+^ MO-DCs migrate to the CNS in response to CCR5 ligands (that is, CCL3, CCL4 and CCL5) amplifying recruitment of CD8^+^ T lymphocytes, initiated by endothelial cells expressing CXCL10 and promote development of ECM.

We provide compelling evidence that acute infection with PbA stimulates differentiation of splenic inflammatory monocytes into MO-DCs in an IFNγ-dependent manner. Malaria-induced MO-DCs possess a strong phagocytic activity, capacity to cross-present antigens to CD8+ T cells and express high levels of CXCL9, CXCL10 and the chemokine receptor CCR5. Hence, splenic CCR5^+^CXCL9/10^+^ MO-DCs seem to play an important role in recruiting and activating T lymphocytes. We hypothesize that after differentiation, splenic CCR5^+^CXCL9/10^+^ MO-DCs migrate to the brain in a CCR5-dependent manner and amplify the recruitment and activation of CXCR3^+^CD8^+^ T cells, promoting neuroinflammation and ECM (Fig. 10).

DCs and inflammatory monocytes play important roles in host resistance to malaria. Indeed, there is a very dynamic distribution of inflammatory monocytes and DC subpopulations in the spleen of acutely infected mice, which changes with the course of infection, parasite virulence and infective *Plasmodium* spp.^5^. For instance, DCs exposed to the erythrocytic stage of *P. chabaudi* produce high levels of IL-12 and promote the production of IFNγ by both T lymphocytes and natural killer cells^14^. This cytokine activates macrophages to produce toxic metabolites, such as reactive nitrogen intermediates and promotes Ig switch to the IgG2a/IgG2c, which mediate host resistance to *Plasmodium* infection in mice^11^,^50^. In addition, both inflammatory monocytes and DCs can internalize and destroy infected RBCs in the spleens and mediate resistance to *P. chabaudi* infection in mice^19^,^21^. However, depending on experimental setting, DCs may become refractory or impaired for presenting antigen to CD4^+^ T and CD8^+^ T cells^51^,^52^. For instance, virulent *P. yoelli* strains impair DC functions to prevent the induction of host protective immune responses^15^. Inflammatory monocytes and DCs also play key roles in the deleterious inflammatory response and pathogenesis of mouse malaria. As examples, DCs promotes cytokinemia and wasting syndrome in *P. chabaudi*-infected mice^22^, and both cDCs and inflammatory monocytes are required for recruitment of CD8^+^ T cells and development of neuroinflammation in the ECM model^23^,^24^,^38^.

Unexpectedly, we found that most CD11c^+^MHC II^high^ cells in the spleen of PbA-infected mice are DC-SIGN^high^CD11b^+^F4/80^+^ consistent with a MO-DC phenotype^35^,^36^. As these cells share various markers with inflammatory monocytes and conventional myeloid, as well as plasmocytoid DCs, some of the existing results in the literature may have missed that a significant proportion of DCs in this mouse malaria model are indeed MO-DCs. Previous studies using the *P. chabaudi* model identified inflammatory monocytes that express low levels of CD11c and a low frequency of CD11c^+^ cells that were CD11b^+^F4/80^+^ (refs 19, 21). Here we provide evidences that in this model, splenic inflammatory monocytes and monocytes are progenitors of MO-DCs. Although the frequency and numbers of MO-DCs cells were markedly increased in the spleen following infection, inflammatory monocytes and monocytes were decreased within the splenic CD11b^+^F4/80^+^ population. Furthermore, we noticed a continuous gradient of MO-DCs expressing Ly6c, from Ly6c^high^ to Ly6c^low^. As inflammatory monocytes are classically Ly6c^high^, we propose that Ly6c^high^MO-DCs are recently differentiated, whereas Ly6c^low^ MO-DCs are fully differentiated. Importantly, we determined here that malaria-induced MO-DCs, but not inflammatory monocytes or monocytes, express high levels of CCR5, CXCL9 and CXCL10, which are all highly relevant for the development of ECM^38^,^39^,^53^.

Depending on TLR or cytokine stimulation, inflammatory monocytes may differentiate into macrophages or MO-DCs, which migrate to lymphoid organs and non-lymphoid tissues^26^,^27^,^28^,^29^,^30^,^35^,^36^. IFNγ not only has an important role in host resistance to *Plasmodium* infection but is also a key mediator of malaria pathogenesis^4^. The high circulating levels of IFNγ elicited during *Plasmodium* infection lead to pro-inflammatory priming^48^,^54^, which promotes systemic inflammation^25^,^48^,^55^. In the case of ECM, treatment with neutralizing anti-IFNγ antibodies or genetic deficiency of IFNγ prevents PbA-induced neuroinflammation and lethality due to ECM^46^,^47^,^56^. Different functions are attributed to IFNγ in the development of ECM, which includes the enhanced production of pro-inflammatory cytokines and chemokines, as well as enhanced expression of adhesion molecules in endothelial cells from brain vasculature^57^,^58^. Important to this study, CXCL9^−/−^, CXCL10^−/−^ and CXCR3^−/−^ mice have a less intense infiltrate of CD8^+^ T cells in the CNS and are more resistant to development of ECM^37^,^38^,^39^. Here we demonstrate that differentiation of MO-DCs is impaired and expression of CXCL9 and CXCL10 abolished in PbA-infected IFNγ^−/−^ or IFNγR^−/−^ mice.

Different studies suggest that TLR7 and TLR9 are important receptors involved in the activation of DCs and initiation of IL-12 and IFNγ production during both human and mouse malaria^6^,^7^,^8^,^9^,^14^,^22^,^59^,^60^. We found that the lysosomotropic compound (E6446), which binds to RNA and DNA, and blocks TLR7 and TLR9 activation, prevents development of ECM^41^,^44^. When given in the first 3 days of infection, treatment with E6446 inhibited CD4^+^ and CD8^+^ T-cell activation and IFNγ production. As a consequence, expression of CXCL9 and CXCL10 by MO-DCs was not induced. These results are consistent with earlier studies showing that IFNγ produced by CD4^+^ T cells induce CXCL9 and CXCL10 expression, and promotes the recruitment of pathogenic CD8^+^ T cells to the brain of PbA-infected mice^38^,^39^,^46^,^47^,^61^,^62^. *Plasmodium* DNA and RNA were also shown to activate other families of cytosolic innate immune receptors. As E6446 binds and ‘inactivates' DNA and RNA that transit through the lysosomes, it may also prevent activation of other DNA/RNA cytosolic sensors that are inducers of type I IFN^42^,^43^,^63^.

We also observed the emergence of CCR5^+^CXCL9/10^+^ MO-DCs in the brain of PbA-infected mice. Among the CD3^−^CD8^−^CD45^high^ haematopoietic cells, most cells expressed CD11b, F4/80, DC-SIGN, Ly6c, MHC II and CD11c^64^,^65^. The increased frequency of CCR5^+^CXCL9/10^+^ MO-DCs coincided with increased expression of CXCL9 and CXCL10 mRNA in the CNS of PbA-infected mice. Although there are evidences that endothelial and other non-haematopoietic cells contribute as sources of CXCL10, we found that among CD45^high^ cells, MO-DCs were the major source of both CXCL9 and CXCL10 (RFP^+^BFP^+^ cells) in the brain of PbA-infected REX3 mice. Hence, CCR5^+^CXCL9/10^+^ MO-DCs are likely to play an important role in recruiting and activating CD4^+^ T and CD8^+^ T lymphocytes during ECM. Recent studies have also proposed that endothelial cells are able to cross-present malaria antigens^66^,^67^. Nevertheless, our data suggest that MO-DCs are key cells presenting malaria antigens to CD8+ T cells in the brain microvasculature and parenchyma.

Interestingly, we found that malaria-induced MO-DCs, but not inflammatory monocytes, express high levels of CCR5. Belnoue *et al*.^53^ suggest an involvement of both brain sequestered leukocytes and non-haematopoietic cells in the CCR5-mediated ECM. As previously described, we found that microglia cells express low levels of CD45 and CCR5 (ref. 68), whereas other non-haematopoietic (CD45^neg^) cells express no CCR5. Importantly, MO-DCs were the main cells expressing high levels of CCR5 in the brain of PbA-infected mice. In addition, deficiency on CCR5 did not have an impact on MO-DCs differentiation in the spleen, but clearly affected their emergence in the brain of PbA-infected mice. Furthermore, our cell transfer experiments show that WT MO-DCs precipitate ECM in PbA-infected CCR5^−/−^ mice. Hence, we favour the hypothesis that CCR5^+^MO-DCs are key mediators of ECM.

A main question raised by our results is whether MO-DCs differentiate in the CNS from BM-derived inflammatory monocytes or are being directly recruited from the pool of splenic CCR5^+^CXCL9/10^+^ MO-DCs. CCL2 and CCR2 play important roles in controlling inflammatory monocyte egress from BM and their migration to the spleen or site of infection^19^,^49^. Consistently, we found an impairment of inflammatory monocytes recruitment to spleens of PbA-infected mice. However, we found that unlike CCR5^−/−^ mice, the frequency of MO-DCs in the brain was only marginally affected and susceptibility to ECM was not altered in CCR2^−/−^ mice infected with PbA. Our results also demonstrate that there is only a small frequency of MO-DCs and CD11b^+^F4/80^+^-expressing CCR5 in the BM from PbA-infected mice, suggesting that inflammatory monocytes/MO-DCs are not being recruited directly from the BM to the brain. Interestingly, we found that despite of impaired recruitment of inflammatory monocytes, there is an increased frequency of CD11b^+^F4/80^+^Ly6c^high^ cells and MO-DCs as well in the spleens of CCR2^−/−^ mice infected with PbA. Thus, we propose that in infected CCR2^−/−^ mice, the splenic monocytes are the precursors cells for MO-DCs.

All three CCR5 chemokine agonists (that is, CCL2, CCL3 and CCL5) are expressed in the brain of PbA-infected mice and may act together to recruit CCR5^+^CXCL9/10^+^ MO-DCs. Although most CXCR3^+^CD8^+^ T cells do not express CCR5, their migration to the CNS is also compromised in infected CCR5^−/−^ mice. These results are consistent with the hypothesis of an amplification loop involving MO-DCs (important source of CXCR3 ligands) and CD8^+^ T lymphocyte (important source of CCR5 ligands)^45^,^69^, resulting in increased CD8^+^ T cell and MO-DC recruitment to the brain in ECM, as recently proposed for Ly6c^+^ monocytic cells and CD8^+^ T cells^24^. Our study provides important novel information regarding the pathogenic mechanism responsible for ECM by demonstrating that the vast majority of Ly6c^+^ that emerge in the CNS of PbA-infected mice are indeed MO-DCs. We also demonstrate how these cells induce T-lymphocyte recruitment to the CNS, as we clearly show that they are an important source of CXCL9 and CXCL10. Finally, we demonstrate that the emergence of these Ly6c^+^ MO-DCs in the CNS is dependent on CCR5 and, surprisingly, independent of CCR2, which is classically involved in inflammatory monocytes recruitment to lymphoid organs and to other sites of infection.

In conclusion, as illustrated in Fig. 10, we propose that immune responses during malaria are initiated by exposure of resident splenic DCs to parasite nucleic acids, which leads to IFNγ production by CD4^+^ T and CD8^+^ T lymphocytes and differentiation of inflammatory monocytes into MO-DCs. The CCR5^+^CXCL9/10^+^ MO-DCs display a potent phagocytic activity and express high levels of MHC II, CD80 and CD86, indicating a great potential not only to recruit, but also to present antigen and activate T lymphocytes. Importantly, CCR5^+^CXCL9/10^+^ MO-DCs become the dominant subset of splenic DCs (CD11c^+^MHC II^high^) and probably play a key role in activating parasite-specific T cells and promoting systemic inflammation during acute malaria. Finally, our results suggest that MO-DCs are not following the conventional path from non-lymphoid tissues to secondary lymphoid organs. Instead, they are being recruited from a pool of splenic MO-DCs to the CNS of PbA-infected mice. Once they reach the brain, the CCR5^+^CXCL9/10^+^ MO-DCs execute their pathogenic role by recruiting and activating CD8^+^ T cells, which are the effector cells in ECM.

## Methods

### Mice

All animals used in this work were 8- to 12-week-old mice. C57BL/6 mice were obtained from either Animal Facility of the UFMG or Jackson Laboratories. The REX3 mouse lineage was generated in C57BL6 embryonic stem cells and crossed with either IFNα/βR^−/−^ or IFNγR^−/−^ to produce the REX3IFNα/βR^−/−^ and REX3IFNγR^−/−^ (ref. 40) in the A.D.L. lab at Massachusetts General Hospital (MGH). The GREAT mice (IFNγ GFP-reporter), the CCR2^−/−^, CCR5^−/−^ and IFNγ^−/−^ mice in the C57BL/6 background were originally purchased from Jackson Laboratories and housed under specific-pathogen-free conditions at MGH or at Oswaldo Cruz Foundation (Fiocruz). All procedures were approved by the MGH Subcommittee on Research and Animal Care and Harvard Committee on Microbiological Safety (IACUC 2005N000176), as well as the Institutional Ethical Committee for Animal Experimentation at Fiocruz (CEUA LW 15-14).

### Infection

The PbA or GFP transgenic PbA (GFP-PbA) kept in liquid nitrogen were thawed and maintained into C57BL/6 mice for up to 12 passages. For experimental infection, PbA iRBCs were collected from donors in heparinized tubes and 1 × 10^5^ iRBCs injected intraperitoneally into naive mice. These mice were observed daily and parasitemia was estimated by counting Giemsa-stained thin blood smears. ECM signs were evaluated by two independent observers using different parameters that included ruffled fur, abnormal postural responses, reduced reflexes, reduced grip strength, coma and convulsions. Mice that demonstrated complete disability in all parameters or died between days 7 and 9 post infection were considered as having ECM^41^.

### Flow cytometry

Brains were removed after intracardial perfusion with 30 ml of PBS; they were minced and the cells were passed through a 70 μm cell strainer and centrifuged on a 45% Percoll for 30 min at 800 *g*. The pelleted cells were washed, diluted 1:1 PBS, counted and stained for flow cytometry. BM cells were obtained by flushing of the bone cavities of the femurs and tibias, then passed through a 70 μm strainer, RBCs lysed and the cells counted by using a haemocytometer. Spleens were removed, passed through a 70 μm strainer, RBCs lysed and splenocytes counted by using a haemocytometer. For flow cytometry analysis, cells were stained with antibodies specific for mouse CD11b (PE-Cy7, clone: M1/70, e-Bioscience, 1:4,000), F4/80 (PE-Cy5, clone: BM8, e-Bioscience, 1:400), CD11c (Alexa 700, clone: N418, e-Bioscience, 1:100), MHC II (phycoerythrin (PE), clone: AF6–120.1, BD, 1:400), DC-SIGN (APC e-fluor 660, clone: MMD3, e-Bioscience, 1:800), Ly6c (e-fluor 450, clone: HK1.4, e-Biosciences, 1:400), CD80 (FITC, clone: 16-10A1, BD, 1:200), CD86 (FITC, clone: GL1, BD, 1:200), CD8 (APC Cy7, clone: 53-6.7, Biolegend, 1:1,000) or (PerCP-Cy 5.5, clone: 53-6.7, e-Bioscience, 1:800), CD3 (Percep Cy5.5, clone: 145-2C11, e-Bioscience, 1:200), CD45 (V500, clone: 30-F11, BD, 1:800), CD62L (PE-Cy7, clone: MEL-14, BD, 1:200), CD44 (Pacific Blue, clone: IM7, Biolegend, 1:200) CD4 (APC, clone: RM4-5, Biolegend, 1:800) at room temperature for 20 min. In REX3 mice, endogenous RFP (CXCL9) and BFP (CXCL10) were read in PE and Pacific Blue channels, respectively. Cytofluorometry was performed by using a Fortessa Cytometer (Becton-Dickinson) and analysed with Flowjo software.

### Phagocytosis assay by flow cytometry

Blood (2 ml) from GFP-PbA-infected and non-infected mice were collected in heparinized tubes. The enrichment of iRBCs was performed using a positive selection kit according to the manufacturer's protocol (Miltenyi Biotec, Bergisch Gladbach, DE). Uninfected RBCs were used as a negative control. The purity was determined by Giemsa-stained thin blood smears. Total splenocytes (5 × 10^5^) from infected (6 days) and non-infected mice were incubated with iRBCs (5 × 10^5^) or RBCs (5 × 10^5^) for 1 h at 37 °C (1:1). Cells were washed and stained with the antibodies specific for mouse CD11b (PE-Cy7), F4/80 (PE-Cy5, 1:400), CD11c (Alexa 700, 1:100), MHC II (PE, 1:400), DC-SIGN (APC e-fluor 660, 1:800) and Ly6c (e-fluor 450, 1:400) at room temperature for 20 min. Flow cytometry was performed by using a Fortessa Cytometer (Becton-Dickinson) and analysed with Flowjo software.

### Cell sorting

Spleens from the C57BL/6 and IFNγ^−/−^ control uninfected and infected mice, at 7 day post infection, were harvested and splenocytes stained with CD11b (PE-Cy7), F4/80 (PE-Cy5), CD11c (Alexa 700), MHC II (PE) and DC-SIGN (APC e-fluor 660), and then submitted to purification by using a cell sorting ARIA (BD). These cells were first gated on FSC-H/FSC-A, to avoid doublets. Next, we first gated on CD11b^+^F4/80^+^ and then on MHC-II^high^DC-SIGN^high^. The gated cells were sorted, collected into fresh new tubes, confirmed to be CD11c^+^ and then frozen in small aliquots of 10,000 cells used for gene expression analysis using the nanostring technology. Alternatively, cells were submitted to cytospin, stained with Giemsa for morphological analysis using corrected optics × 40 and × 100 (oil) in an optical microscope (Olympus, GXML3200B).

### Scanning electronic microscopy

After cell sorting, aliquots of MO-DCs were fixed in 2.5% buffered glutaraldehyde solution, 0.1 M pH 7.2, 6 h, 8 °C. Cells were then washed with the same buffer and fixed in a mixture of 1% osmium tetroxide and 1.5% (w/v) potassium ferrocyanide, dehydrated in a graded series of ethanol, infiltrated and embedded in Araldite 502 (Electron Microscopy Sciences, Hatfield, PA, USA). Sections were stained with 2% uranyl acetate and Reynolds lead citrate and then analysed using SEM (DSM 960A).

### Antigen presentation

Cell trace-labelled CD8^+^ T cells specific for OVA (OT-I) were cultured with sorted monocytes or MO-DCs from the spleens of PbA-infected mice at different APC/T cell ratio (1:3, 1:10 and 1:30) in the presence of 40 μg ml^−1^ of OVA protein (OVA grade V, Sigma-Aldrich). For MO and MO-DC sorting, cells from the spleens 5 days after PbA infection were pre-enriched using anti-CD11b MACS beads and LS MACS Separation Columns (Miltenyi Biotec). MO-DCs and monocytes were then sorted as CD11b^hi^F4/80^hi^DC-SIGN^hi^MHCII^+^CD11c^−^ and CD11b^hi^F4/80^hi^DC-SIGN^−^MHCII^−^CD11c^−^, respectively. Splenic naive OT-I transgenic T cells were pre-enriched by negative selection using Mouse CD8^+^ T Cell Isolation Kit (Stemcell Technologies) and then sorted as CD8^+^CD62^hi^CD44^low/−^. Sorted naive OT-I cells were labelled with 1.25 μM of Cell Trace Violet (Invitrogen) and added to 96-well round-bottom plate at 30,000 per well. After 3 days, OVA-specific proliferation of live (Fixable Viability Dye negative, eBioscience) OT-I cells was evaluated by Cell Trace Violet dilution and staining with monoclonal antibody to CD8. Sandwich kit ELISA (Biolegend) was used to measure IFNγ in the supernatant co-cultures of OT-I and monocytes or MO-DCs according to the manufacturer's recommendations.

### Confocal

Spleens were harvested into PLP buffer ([0.05 M phosphate buffer containing 0.2 M L-lysine pH 7.4, 2 mg ml^−1^ NaIO_4_ and 10 mg ml^−1^ paraformaldehyde), fixed for 5–12 h and dehydrated in 30% sucrose before embedding in OCT freezing media (Sakura Fineteck). Ten-millimetre frozen sections were cut on a CM3050S cryostat (Leica). Sections were dried for 2 h, acetone fixed for 10 min, rehydrated, fixed with formaldehyde-calcium solution and washed with PBS. Sections were blocked in PBS containing American Hamster or Rat gamma globulin (100 μg ml^−1^), stained in PBS, F4/80 (APC) or CD11c (APC) and embedded in prolong gold. Images were acquired on a LSM510 confocal microscope (Carl Zeiss Mircoimaging).

### Adoptive cell transfers

CD11b^+^F4/80^+^ cells were enriched from the spleens of uninfected CD45.2^+^ donor mice. Spleen cells were incubated with a mix of PE-labelled antibodies: CD4 (clone: GK1.5, eBioscience), CD8 (clone: 53-6.7, eBioscience), TCR-β (clone: H57–597, eBioscience), CD19 (clone: 1D3, eBioscience), Ly6G (clone: 1A8, eBioscience), Ly49 (clone: YLI-90, eBioscience) and CD11c (clone: N418, eBioscience), and monocytes were enriched up to 40–60 × using the PE-multisort kit (Miltenyi Biotec 130-090-757) for negative selection. Enriched monocytes (∼2 × 10^6^ cells) were injected intravenously (i.v.) into 6-week-old, non-irradiated CD45.1^+^ recipient mice uninfected or 3 days post infection with PbA. After 48 h, spleen cells were obtained and analysed by flow cytometry and the frequency of MO-DCs, inflammatory monocytes and monocytes from CD45.2^+^ donor and CD45.1^+^ recipient mice was determined. Alternatively, MO-DCs were enriched from the spleens of either WT or CCR5^−/−^ at 5 days post infection by consecutive positive selection of CD11b^+^ (catalogue number 130-097-142) and then CD11c^+^ (catalogue number 130-052-001) cells using the MicroBeads kit (Miltenyi Biotec, CD11b). MO-DCs were at least 80% pure after purification. Three million WT or CCR5^−/−^ MO-DCs were then transferred i.v. to CCR5^−/−^ mice at 4 days post infection with PbA. Mice were checked daily for signs of ECM and survival.

### Intravital confocal microscopy

MO-DCs were purified from the spleens of PbA-infected from four mice by consecutive positive selection of CD11b^+^ (catalogue number 130-097-142) and then CD11c^+^ (catalogue number 130-052-001) cells using the MicroBeads kit (Miltenyi Biotec, CD11b). The cells were then incubated with CFSE (2 μM per 1 × 10^6^ cells in 100 μl) for 8 min and washed. The vasculature was stained using 10 μl of a PE-coupled anti-CD31 antibody stock solution (0.2 mg ml^−1^). Infected mice (5 days post-infection) were anaesthetized intraperitoneally (100 μl for each animal) with a mixture of ketamine (37.5 mg ml^−1^, final concentration) and xylazine (2.5 mg ml^−1^, final concentration). After anaesthetization, one million of CFSE-stained CD11b^+^CD11c^+^ cells and anti-CD31 were injected i.v. and a cranial window (2 × 2 mm) was drilled above the parietal cortex, leaving the dura mater intact to exposed and visualize the brain vasculature. Mouse was immobilized using the stereotaxis apparatus, and CD11b^+^ CD11c^+^ cells and endothelium were observed using a confocal microscope (Nikon, ECLIPSE 50i, × 20 objective lens) outfitted with a fluorescent light source (epi-illumination at 510–560 nm, using a 590 nm emission filter). The number of migrating cells was determined offline during the video playback analyses. In each animal, a field was recorded and analysed, to determine the total number of cells. Quantitative image analysis was performed by using the software Volocity (Version 6.3).

### DC migration assay

Splenic CD11b^+^CD11c^+^ cells from uninfected and 6 days postinfection mice were purified using a positive selection kit, as described in the intravital confocal microscopy section (Miltenyi Biotec). Cells were stained with anti-CD11b (PE-Cy7, clone: M1/70, e-Bioscience) and anti-CD11c (Alexa 700, clone: N418, e-Bioscience), and purity checked by FACS analysis. CD11b^+^CD11c^+^ cells were suspended in RPMI medium plus 10% fetal bovine serum at 1 × 10^5^ cells per ml. To assess the migratory capacities of MO-DCs cells, we used a 12-well Transwell microplate (Corning, Amboise, FR) with 5 μm membrane pore size that forbade the passive diffusion, but allowed the active migration of MO-DCs. For each condition tested, lower chambers of the transwell were filled with 600 μl in the absence or presence of 0.1, 1 or 10 ng ml^−1^ CCL2 (Biolegend) and/or CCL5 (e-bioscience). MO-DCs (1 × 10^5^ in 100 μl) were deposited in the upper chamber of the Transwell and were allowed to migrate for 3 h at 37 °C. Migrating DCs were harvested from the lower chamber and were counted using Neubauer chamber. Enumerations were performed twice, to assess the reliability of the method.

### Cytokine assays

The levels of IFNγ were measured in sera of control and infected mice by using the Cytometric Bead Array Mouse Th1/Th2/Th17 Cytokines Kit (BD Becton-Dickinson, catalogue number 560485)^22^.

### Quantitative PCR

Total RNA from spleens and brains from uninfected or PbA-infected mice was extracted by using TRIzol and complementary DNA prepared using Multiscribe RT (Applied Biosystems). Quantitative PCR was performed with SYBR Green Master Mix (Applied Biosystems) using a Realplex2 mastercycler (Eppendorf)^1^. Primer sequences for measuring *IFNγ* and chemokine gene expression were: *CXCL9,* 5′-AATGCACGATGCTCCTGCA-3′ and 5′-AGGTCTTTGAGGGATTTGTAGTGG-3′; *CXCL10*, 5′-GCCGTCATTTTCTGCCTCA-3′ and 5′-CGTCCTTGCGAGAGGGATC-3′; *IFNγ,* 5′-AACGCTACACACTGCATCTTGG-3′ and 5′-GCCGTGGCAGTAACAGCC-3′; *CD8*, 5′-GACGAAGCTGACTGTGGTTGA-3′ and 5′-GCAGGCTGAGGGTGGTAAG-3′; *CCL2*, 5′-TGGCTCAGCCAGATGCAGT-3′ and 5′-TTGGGATCATCTTGCTGGTG-3′; *CCL3*, 5′-CCAAGTCTTCTCAGCGCCAT-3′ and 5′-TCCGGCTGTAGGAGAAGCAG-3′; *CCL4*, 5′-TCTTGCTCGTGGCTGCCT-3′ and 5′-GGGAGGGTCAGAGCCCA-3′; and *CCL5*, 5′-CAAGTGCTCCAATCTTGCAGTC-3′ and 5′-TTCTCTGGGTTGGCACACAC-3′. All samples were normalized using *β2-MICROGLOBULIN* or glyceraldehyde 3-phosphate dehydrogenase expression employing either 5′-CCGAACATACTGAACTGCTACGTAA-3′ and 5′-CCCGTTCTTCAGCATTTGGA-3′ or 5′-GTGGTGAAGCAGGCATCTGA-3′ and 5′-GGGAGTCACTGTTGAAGTCGC-3′ primers, respectively.

### Quantification of RNA expression by nanostring

Purified MO-DCs from C57BL/6 and *IFNγ*^−/−^ infected with PbA were analysed by NanoString methodology. Briefly, 10,000 cells were resuspended in 1.0 μl of Qiagen RLT lysis buffer and hybridized to the target-specific code set on 65 °C. The code set contained probes against a panel of 561 genes encoding relevant innate immunity proteins (nCounter Mouse Immunology). After incubation, samples (three animals per group) were loaded onto the NanoString Prep station for excess reporter removal, binding to cartridge surface and probe scanning. After scanning and data collection onto a digital analyser, data normalization was performed against positive and negative control oligonucletides and 14 housekeeping genes. Normalized results are represented as the relative mRNA level. The fold change was defined by rate of mRNA's numbers of copies of the infected by non-infected mice. The software Multiple Experiment Viewer was used to generate the heat maps. Differences in gene expression were considered significant if *P*<0.05 as defined by unpaired *t*-test.

### Drug and treatment

TLR7/9 antagonist (E6446, Eisai Pharmaceuticals) was dissolved in water and mice were treated 120 mg kg^−1^ per day from a day before infection to the indicated day post infection, given in 100 μl through oral gavage once daily^41^. Mice treated with vehicle (water) were used as a control.

### Statistical analysis

All data were analysed using Graphpad Prism 6.0 Software. Two-tailed Student's *t*-tests were used for data analysis and generation of *P*-values. Mann–Whitney testing was used for non-parametric analysis when data did not fit a Gaussian distribution. Analysis of variance via post-Tukey's test for multiple comparisons was used for comparing more than two samples. A *P*<0.05 value was considered statistically significant. All data are represented as median with individual data points representing individual samples. Bar graph data show s.d. error bars.

### Data availability

The authors declare that the data supporting the findings of this study are available within the article and its Supplementary Information files.

## Additional information

**How to cite this article:** Hirako, I. C. *et al*. Splenic differentiation and emergence of CCR5^+^CXCL9^+^CXCL10^+^ monocyte-derived dendritic cells in the brain during cerebral malaria. *Nat. Commun.* **7,** 13277 doi: 10.1038/ncomms13277 (2016).

**Publisher's note:** Springer Nature remains neutral with regard to jurisdictional claims in published maps and institutional affiliations.

## Supplementary Material

Supplementary Information Supplementary Figures 1-9

Supplementary Movie 1 In vivo imaging of WT MO-DCs in the brain of CR7BL/6mice infected with PbA. MO-DC enriched populations were obtained from C57BL/ mice 5 days postinfection labeled with CSFE and transferred i.v. (one million cells/mouse) to PbA-infected WT mice. The presence of CSFE labeled cells were detected in the microvasculature and brain parenchyma by confocal microscopy 15-30 minutes after cell transfer in the orbital vein.

Supplementary Movie 2 In vivo imaging of WT MO-DCs in the brain of CR7BL/6mice infected with PbA. MO-DC enriched populations were obtained from C57BL/ mice 5 days postinfection labeled with CSFE and transferred i.v. (one million cells/mouse) to PbA-infected WT mice. The presence of CSFE labeled cells were detected in the microvasculature and brain parenchyma by confocal microscopy 15-30 minutes after cell transfer in the orbital vein.

Supplementary Movie 3 In vivo imaging of CCR5-/-MO-DCs in the brain of C57BL/6 mice infected with PbA. MO-DC enriched populations were obtained from CCR5-/- mice at 5 days post-infection labeled with CSFE and transferred i.v. (one million cells/mouse) to PbA-infected WT mice. The presence of CSFE labeled cells were detected in the microvasculature and brain parenchyma by confocal microscopy 15-30 minutes after cell transfer in the orbital vein.

Supplementary Movie 4 In vivo imaging of CCR5-/-MO-DCs in the brain of C57BL/6 mice infected with PbA. MO-DC enriched populations were obtained from CCR5-/- mice at 5 days post-infection labeled with CSFE and transferred i.v. (one million cells/mouse) to PbA-infected WT mice. The presence of CSFE labeled cells were detected in the microvasculature and brain parenchyma by confocal microscopy 15-30 minutes after cell transfer in the orbital vein.

Supplementary Movie 5 In vivo imaging of CCR5-/-MO-DCs in the brain of C57BL/6 mice infected with PbA. MO-DC enriched populations were obtained from CCR5-/- mice at 5 days post-infection labeled with CSFE and transferred i.v. (one million cells/mouse) to PbA-infected WT mice. The presence of CSFE labeled cells were detected in the microvasculature and brain parenchyma by confocal microscopy 15-30 minutes after cell transfer in the orbital vein.

Supplementary Movie 6 In vivo imaging of CCR5-/-MO-DCs in the brain of C57BL/6 mice infected with PbA. MO-DC enriched populations were obtained from CCR5-/- mice at 5 days post-infection labeled with CSFE and transferred i.v. (one million cells/mouse) to PbA-infected WT mice. The presence of CSFE labeled cells were detected in the microvasculature and brain parenchyma by confocal microscopy 15-30 minutes after cell transfer in the orbital vein.
