# Supplementary material for: Splenic differentiation and emergence of CCR5+CXCL9+CXCL10+ monocyte-derived dendritic cells in the brain during cerebral malaria
Source: Nat Commun. 2016 Nov 3;7:13277. doi: 10.1038/ncomms13277 (PMC5097164; doi:10.1038/ncomms13277)
Supplement: Supplementary Information — Supplementary Figures 1-9 [file ncomms13277-s1.pdf]

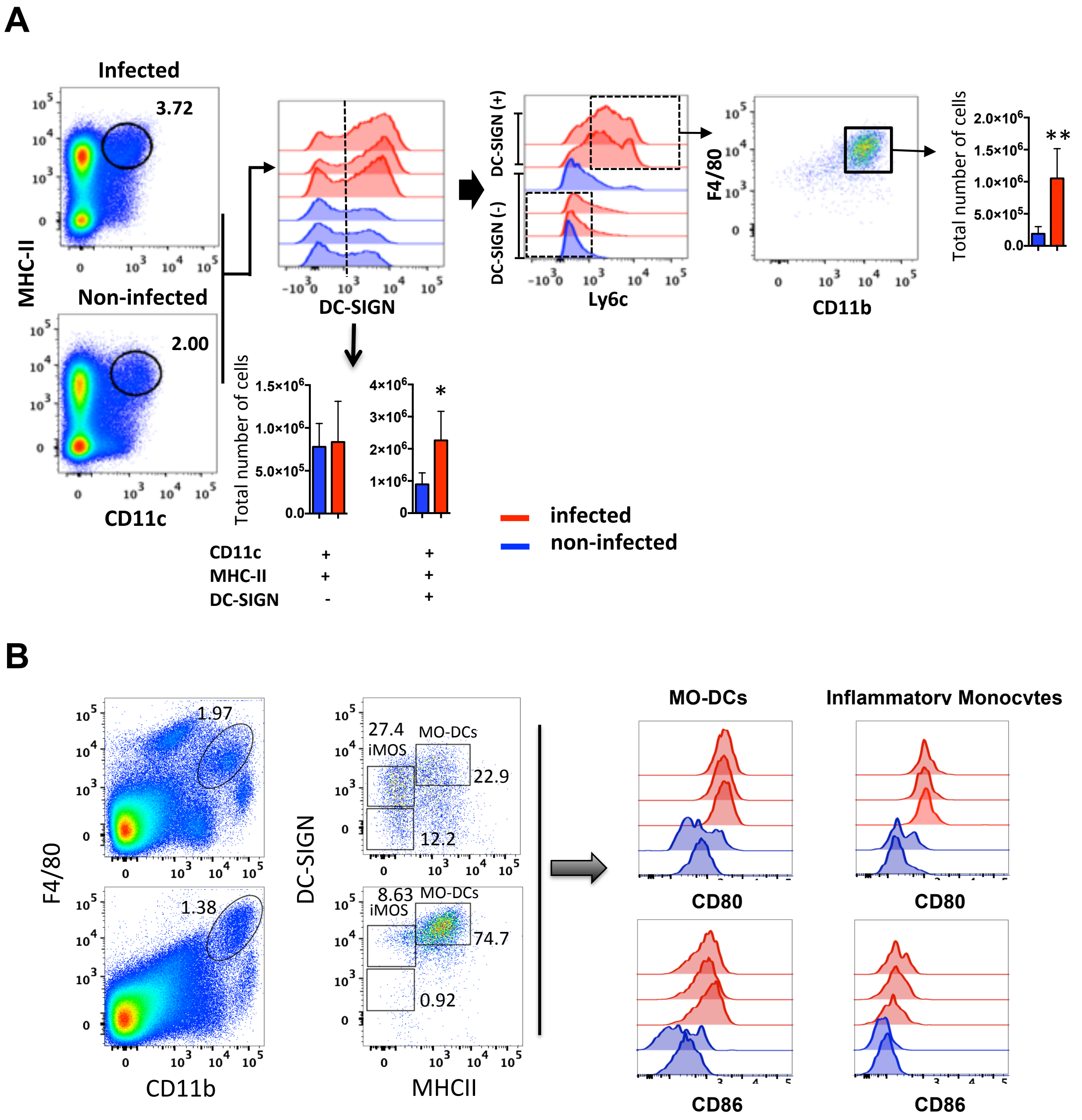

Supplementary Figure 1. Immunophenotyping of MO-DCs. Splenocytes were harvested from control and infected mice at 5 days post-infection. **(A)** Splenocytes were first gated for CD11c^{+}MHC II^{high} cells and then for DC-SIGN^{high} and Ly6c^{+} cells. Over 90% of CD11c^{+}MHC II^{high}DC-SIGN^{high}Ly6c^{+} cells were CD11b^{+}F4/80^{+}. Bar graphs correspond to total number of cells (four mice per group). Results are expressed as median ± s.d. Differences considered statistically significant between infected and non-infected, when * p<0.05 after Mann-Whitney U Test analysis. The data shown are representative of five independent experiments. **(B)** Levels of CD80 and CD86 expression in inflammatory monocytes (CD11b^{+}F4/80^{+}DC-SIGN^{low}MHCII^{-}) and MO-DCs (CD11b^{+}F4/80^{+}DC-SIGN^{high}MHCII^{high}) from control and infected mice. Representative results of four mice per group of control versus infected mice.

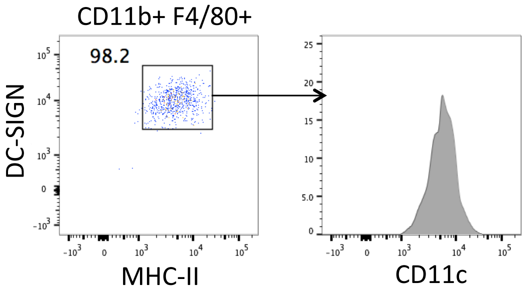

Supplementary Figure 2. Purity of MO-DCs isolated by cell sorting. Spleens were harvested at 6 days after infection with PbA. MO-DCs were labeled with F4/80$^+$CD11b$^+$DC-SIGN^{high}MHC II^{high} cells and submitted to sorting by flow cytometry. We obtained over 98.2% purity of cells that were also CD11c$^+$. The data shown are representative of MO-DCs purified from at least ten mice and used for analysis by optical and scanning electronic microscopy **(Figure 1)** as well as gene expression by nanostring **(Figures 2 and 4).**

A

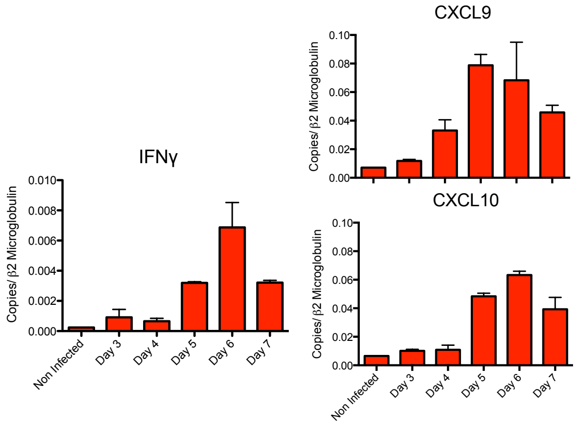

B

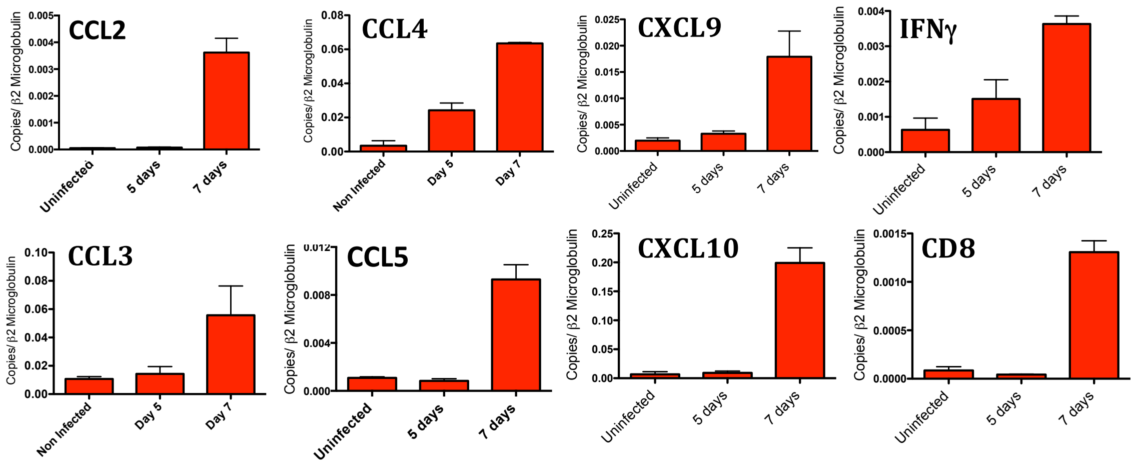

Supplementary Figure 3. Kinetics of *IFNγ*, *CD8*, and chemokine mRNA expression in spleens and brains from PbA infected mice. (**A)** Spleens were harvested at 0 and 3 to 7 days post-infection. (**B)** Brains were harvested at days 0, 5 and 7 post-infection with PbA. Total RNA was extracted from individual spleens and brains and levels of cytokine/chemokine mRNA analyzed by qPCR and normalized by *GAPDH* or *β2-MICROGLOBULIN* expression. The data are representative of two independent experiments with four mice per group (values are means ± s.d) .

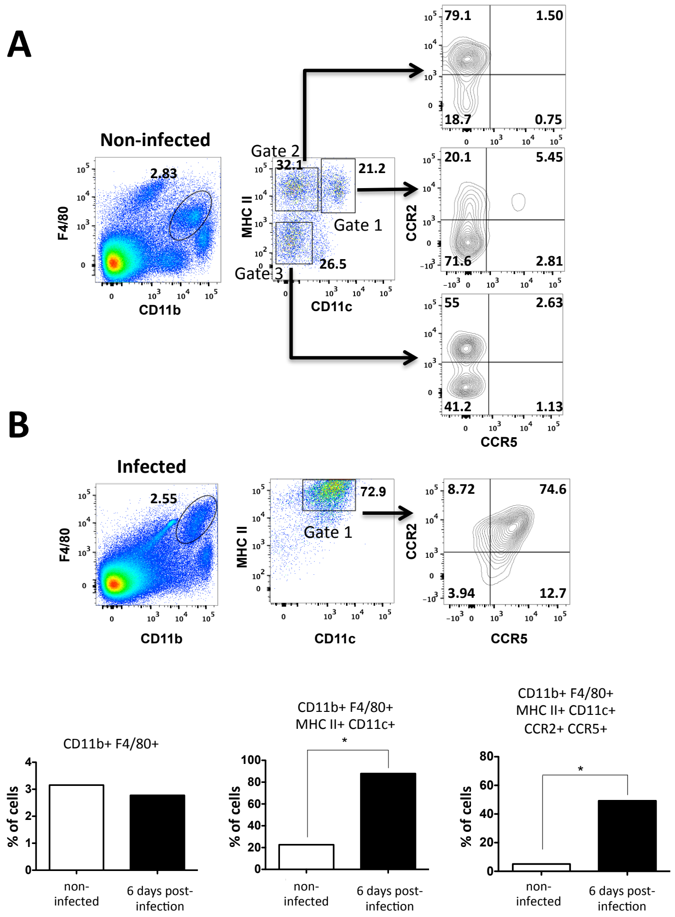

Supplementary Figure 4. CCR5 expression by splenic MO-DCs from C57BL6 mice infected with PbA. (A) Representative primary flow cytometric dot plots of spleens from uninfected control mice are shown. The percentage of MO-DCs (Gate 1, F4/80$^+$/CD11b$^+$DC-SIGN$^+$MHC II$^+$), Gate 2 (F4/80$^+$/CD11b$^+$DC-SIGN$^+$MHC II$^-$), and Gate 3 (F4/80$^+$/CD11b$^+$DC-SIGN$^-$MHC II$^-$) expressing CXCR2 and CXCR5 are indicated. **(B)** C57BL/6 mice were inoculated with PbA, and spleens harvested 6 days later. Representative primary flow cytometric dot plots of spleens from infected mice are shown. The percentage of MO-DCs (Gate 1, F4/80$^+$/CD11b$^+$DC-SIGN$^+$MHC II$^+$) expressing CXCR2 and CXCR5 are indicated. **(C)** Frequency of splenic MO-DCs and other cell populations in controls and infected C57BL/6 are shown in bar graphs. This is one representative of three independent experiments with three or four mice per group. Results are expressed as median. An asterisk indicates that difference is statistically significant when comparing infected and non-infected mice (p<0.01) after Mann-Whitney U test (values are medians ± s.d) .

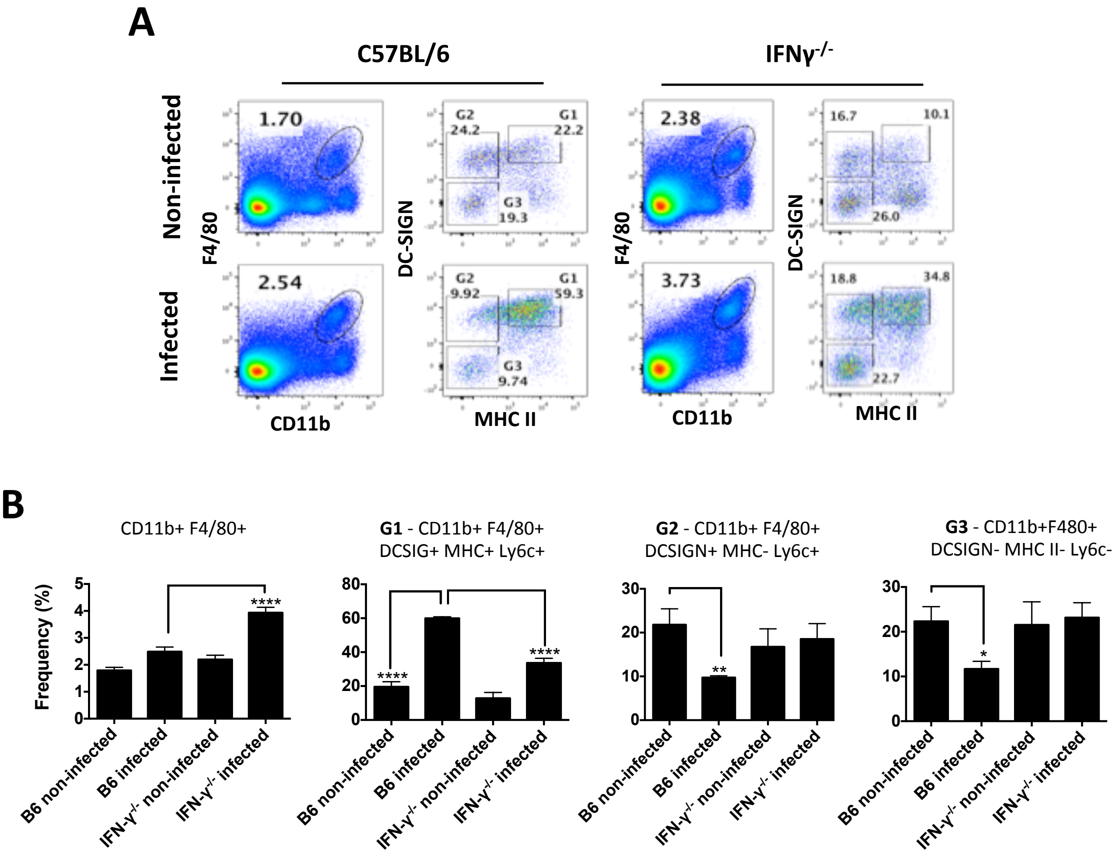

Supplementary Figure 5. Frequency of splenic MO-DCs is reduced in *IFNγ*$^{-/-}$ mice infected with PbA. C57BL/6 and *IFNγ*$^{-/-}$ mice were inoculated with PbA, and spleens harvested 6 days later. **(A)** Representative primary flow cytometric dot plots of spleens from infected mice are shown. The percentage of MO-DCs (Gate 1, F4/80^{+}/CD11b^{+}DC-SIGN^{+}MHC II^{+}), Gate 2 (F4/80^{+}/CD11b^{+}DC-SIGN^{+}MHC II^{-}), and Gate 3 (F4/80^{+}/CD11b^{+}DC-SIGN^{-}MHC II^{-}) are indicated. (**B)** Frequency of splenic MO-DCs and other cell populations in controls and infected C57BL/6 and *IFNγ*$^{-/-}$ mice are shown in bar graphs. The average and SD are one representative of two independent experiments with three or four mice per group. Differences were considered statistically significant when * p<0.05, ** p<0.01, **** and p<0.0001 as indicated by two-way ANOVA analysis (values are means ± s.d.) .

A

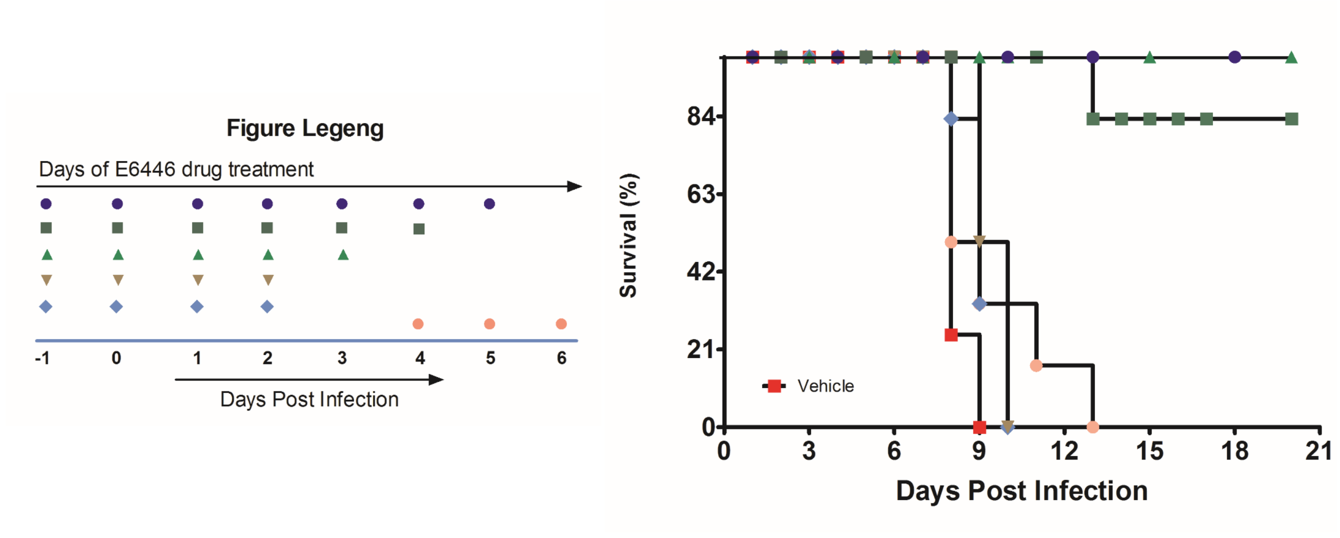

B

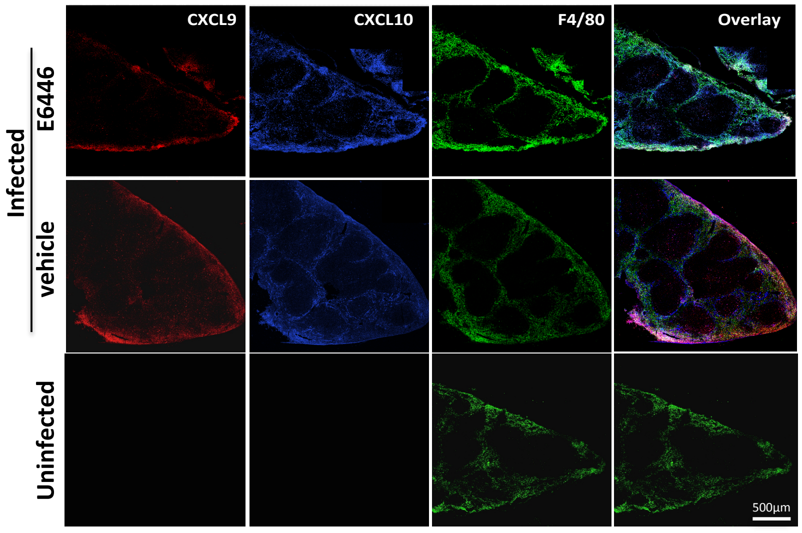

Supplementary Figure 6. Treatment with E6446 prevents lethality expression of CXCL9 and CXCL10 by splenocytes in PbA infected mice. (A) Mice were treated using different schedules (left panel) and followed for survival (right panel). The selected regimen (day -1 to day 3 post-infection) was used in all experiments presented in this study. **(B)** Illustrative confocal analysis of spleens from uninfected and infected REX3 mice treated with E6446. Treatment with E6446 inhibits expression of RFP (CXCL9) and BFP (CXCL10) by spleen cells from PbA-infected REX3 mice (scale bar, 500 µm)

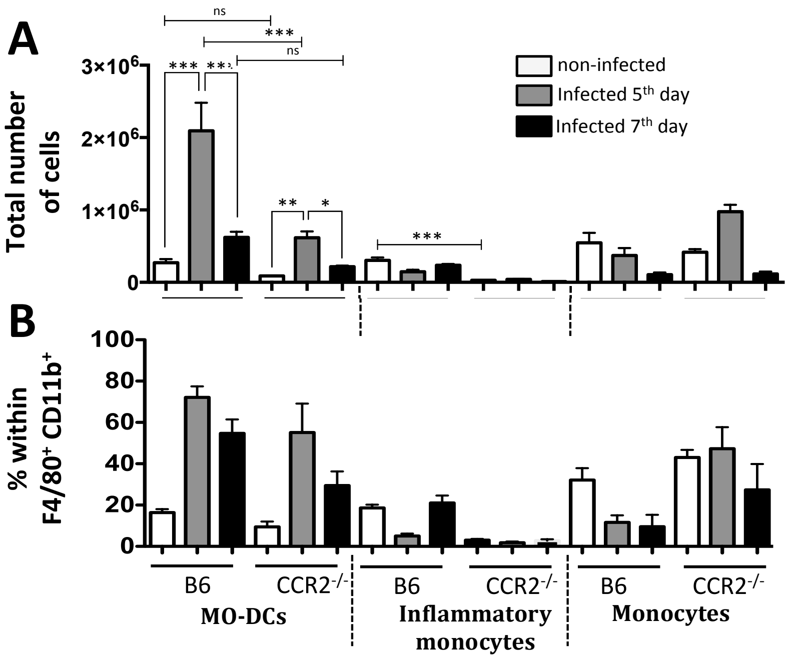

Supplementary Figure 7. Frequency and total numbers of MO-DCs in spleens from *CCR2$^{-/-}$* mice infected with PbA. C57BL/6 and *CCR2$^{-/-}$* mice were infected with PbA and spleens harvested at 0, 5 and 7 days later. Splenocytes were gated on CD11b and F4/80 and then analyzed for DC-SIGN and MHC II expression. Average and standard deviation of **(A)** total number and **(B)** frequency of MO-DCs (Gate 1, F4/80$^+$/CD11b$^+$DC-SIGN$^+$MHC II$^+$), Gate 2 (F4/80$^+$/CD11b$^+$DC-SIGN$^+$MHC II$^-$), and Gate 3 (F4/80$^+$/CD11b$^+$DC-SIGN$^-$MHC II$^-$) within CD11b$^+$F4/80$^+$ cells are shown. The presented data are representative of one out of two experiments with four mice per group. Differences were considered statistically significant when * $p<0.05$,**$p<0.01$ and ***$p<0.001$ as indicated by two-way ANOVA analysis (values are means ± s.d.).

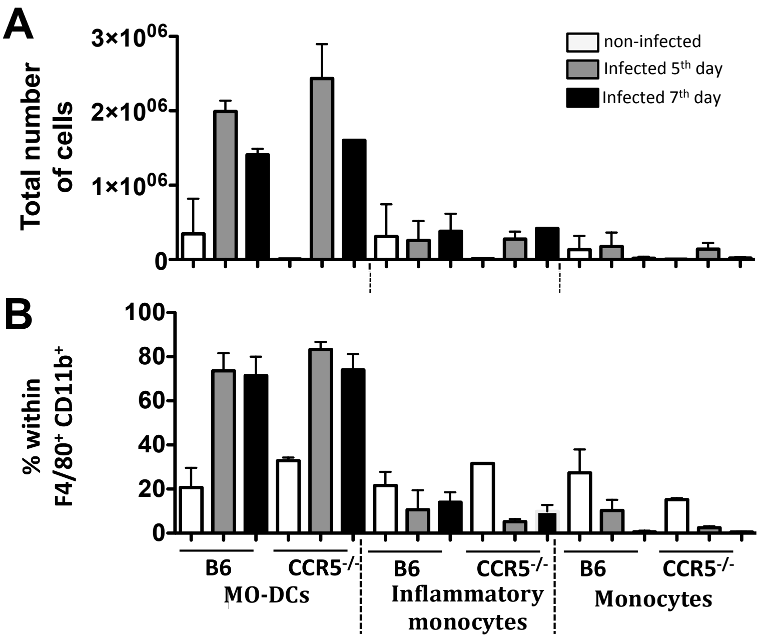

Supplementary Figure 8. Frequency and total number of splenic MO-DCs is not affected in *CCR5$^{-/-}$* mice infected with PbA. C57BL/6 and *CCR5$^{-/-}$* mice were inoculated with PbA, and spleens harvested 0, 5 and 7 days later. Splenocytes were gated on CD11b and F4/80 and then analyzed for DC-SIGN and MHC II expression. Average and standard deviation of **(A)** total number and **(B)** frequency of MO-DCs (Gate 1, F4/80$^+$/CD11b$^+$DC-SIGN$^+$MHC II$^+$), Gate 2 (F4/80$^+$/CD11b$^+$DC-SIGN$^+$MHC II$^-$), and Gate 3 (F4/80$^+$/CD11b$^+$DC-SIGN$^-$MHC II$^-$) within CD11b$^+$F4/80$^+$ cells are shown. The presented data are representative of one out of two experiments with four mice per group. Differences were considered statistically significant when * $p<0.05$,**$p<0.01$ and ***$p<0.001$ as indicated by two-way ANOVA analysis (values are means ± s.d.).

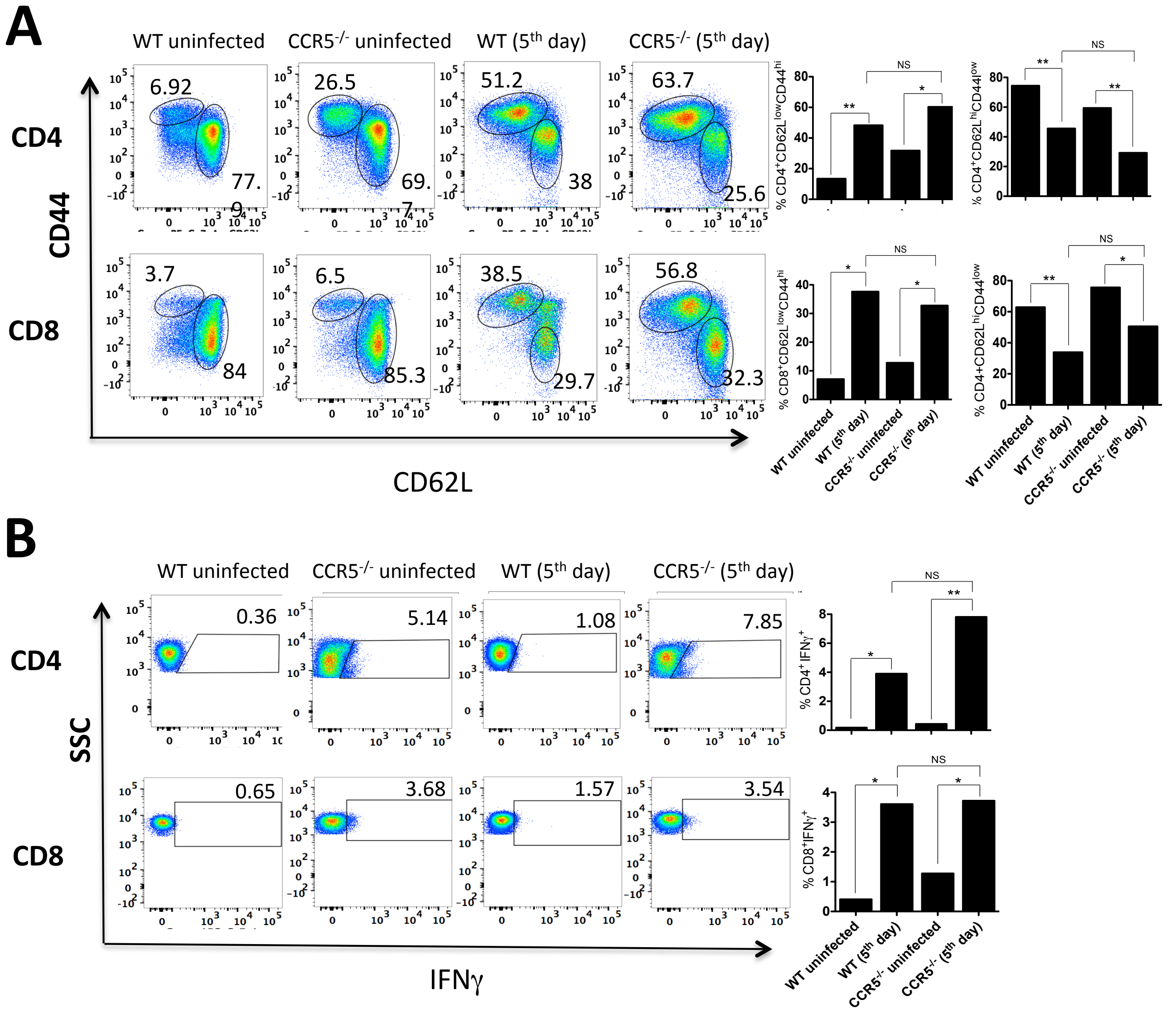

Supplementary Figure 9. Unimpaired T cell activation in *CCR5*$^{-/-}$ mice infected with PbA. C57BL/6 and *CCR5*$^{-/-}$ mice were inoculated with PbA, and spleens harvested 5 days later. Splenocytes were gated on CD4 or CD8 and then analyzed for CD44, CD62L or IFNγ expression. **(A)** Bar graphs correspond to the percentage of naïve (CD62L^{high}CD44^{low}) versus activated effector (CD62L^{low}CD44^{high}) CD4^{+} T (top panels) and CD8^{+} T (bottom panels) cells. **(B)** Splenocytes from uninfected and infected mice were stimulated with PMA (50 ng/ml) and ionomycin (500 ng/ml) for 4h in culture containing brefeldin A, and analyzed by flow cytometry. Bar graphs correspond to the median of percentage of **(A)** naïve vs activate or **(B)** IFNγ production from CD4^{+} T (top panels) and CD8^{+} T (bottom panels) cells. Differences were considered statistically significant when * p<0.05 or **p<0.01 as indicated by one-way ANOVA analysis.
